# Distinct neural signatures detected for ADHD subtypes after controlling for micro-movements in resting state functional connectivity MRI data

**DOI:** 10.3389/fnsys.2012.00080

**Published:** 2013-02-04

**Authors:** Damien A. Fair, Joel T. Nigg, Swathi Iyer, Deepti Bathula, Kathryn L. Mills, Nico U. F. Dosenbach, Bradley L. Schlaggar, Maarten Mennes, David Gutman, Saroja Bangaru, Jan K. Buitelaar, Daniel P. Dickstein, Adriana Di Martino, David N. Kennedy, Clare Kelly, Beatriz Luna, Julie B. Schweitzer, Katerina Velanova, Yu-Feng Wang, Stewart Mostofsky, F. Xavier Castellanos, Michael P. Milham

**Affiliations:** ^1^Department of Behavioral Neuroscience, Advanced Imaging Research Center, Oregon Health and Science UniversityPortland, OR, USA; ^2^Department of Psychiatry, Advanced Imaging Research Center, Oregon Health and Science UniversityPortland, OR, USA; ^3^Department of Computer Science and Engineering, Indian Institute of Technology RoparRupnagar, Punjab, India; ^4^Department of Neurology, Washington UniversitySt. Louis, MO, USA; ^5^Phyllis Green and Randolph Cowen Institute for Pediatric Neuroscience, NYU Langone Medical CenterNew York, NY, USA; ^6^Department of Cognitive Neuroscience, Radboud University Nijmegen Medical CenterNijmegen, Netherlands; ^7^PediMIND Program, Bradley Hospital, Brown University School of MedicineEast Providence RI, USA; ^8^University of Massachusetts Medical CenterWorcester, MA, USA; ^9^Department of Psychiatry, University of PittsburghPA, USA; ^10^Departments of Psychiatry, Behavioral Sciences, and the MIND Institute, University of California Davis School of MedicineSacramento, CA, USA; ^11^Institute of Mental Health, Peking UniversityBeijing, China; ^12^Key Laboratory of Mental Health, Ministry of Health, Peking UniversityBeijing, China; ^13^Kennedy Krieger InstituteBaltimore, MD, USA; ^14^Johns Hopkins UniversityBaltimore, MD, USA; ^15^Nathan Kline InstituteOrangeburg, NY, USA; ^16^Center for the Developing Brain, Child Mind InstituteNew York, NY, USA

**Keywords:** ADHD, functional connectivity, support vector machines, RDoC, research domain criteria

## Abstract

In recent years, there has been growing enthusiasm that functional magnetic resonance imaging (MRI) could achieve clinical utility for a broad range of neuropsychiatric disorders. However, several barriers remain. For example, the acquisition of large-scale datasets capable of clarifying the marked heterogeneity that exists in psychiatric illnesses will need to be realized. In addition, there continues to be a need for the development of image processing and analysis methods capable of separating signal from artifact. As a prototypical hyperkinetic disorder, and movement-related artifact being a significant confound in functional imaging studies, ADHD offers a unique challenge. As part of the ADHD-200 Global Competition and this special edition of Frontiers, the ADHD-200 Consortium demonstrates the utility of an aggregate dataset pooled across five institutions in addressing these challenges. The work aimed to (1) examine the impact of emerging techniques for controlling for “micro-movements,” and (2) provide novel insights into the neural correlates of ADHD subtypes. Using support vector machine (SVM)-based multivariate pattern analysis (MVPA) we show that functional connectivity patterns in individuals are capable of differentiating the two most prominent ADHD subtypes. The application of graph-theory revealed that the Combined (ADHD-C) and Inattentive (ADHD-I) subtypes demonstrated some overlapping (particularly sensorimotor systems), but unique patterns of atypical connectivity. For ADHD-C, atypical connectivity was prominent in midline default network components, as well as insular cortex; in contrast, the ADHD-I group exhibited atypical patterns within the dlPFC regions and cerebellum. Systematic motion-related artifact was noted, and highlighted the need for stringent motion correction. Findings reported were robust to the specific motion correction strategy employed. These data suggest that resting-state functional connectivity MRI (rs-fcMRI) data can be used to characterize individual patients with ADHD and to identify neural distinctions underlying the clinical heterogeneity of ADHD.

## Introduction

Brain imaging has increasingly become a useful tool in modern medicine. Most notably, magnetic resonance imaging (MRI) has emerged as an accurate and reliable approach to identifying abnormalities characteristic of congenital, neoplastic, ischemic, inflammatory, metabolic, and infectious processes in the brain. Unfortunately, the clinical utility of imaging is markedly diminished when considering conditions that are not accompanied by gross structural or inflammatory abnormalities. Chronic pain syndromes, movement disorders, and, in particular, psychiatric illnesses, have thus far remained without scientifically founded clinical benefit from the introduction of brain imaging into medical practice (Matthews et al., 2006). After two decades of development, there is now growing enthusiasm that functional MRI could achieve clinical utility for a broad range of neuropsychiatric disorders.

This growing enthusiasm stems, in part, from the emergence of resting-state functional connectivity MRI (rs-fcMRI). Originally described by Biswal et al. (1995), rs-fcMRI is based on the discovery that spontaneous neural activity (Biswal et al., 1995; Leopold et al., 2003; Nir et al., 2006; Scholvinck et al., 2010) is associated with correlated low-frequency (<~0.1 Hz) blood oxygen level-dependent (BOLD) signal fluctuations in functionally related brain regions at rest (Biswal et al., 1995). By cross-correlating the time series of a particular brain region (seed region) with other regions or voxels, one can determine which regions are “functionally connected.” Importantly, rs-fcMRI can be used during sleep as well as during sedation (Fukunaga et al., 2006, 2008; Vincent et al., 2007; Greicius, 2008; Horovitz et al., 2008); it yields consistent results across subjects, scans, and days (van de Ven et al., 2004; Damoiseaux et al., 2006; Shehzad et al., 2009; Van Dijk et al., 2010), and rs-fcMRI results are remarkably reliable across imaging centers (Biswal et al., 2010). These features make rs-fcMRI an attractive measure for translational and clinical applications. As highlighted in the ADHD-200 competition (ADHD-200-Consortium, 2012), and this special edition highlighting the work of the competitors, the race for these sorts of applications is currently underway (ADHD-200-Consortium, 2012).

Despite the enthusiasm, two key rate-limiting steps remain for the advancement of functional neuroimaging approaches in the clinical realm. First is the acquisition of large-scale datasets capable of clarifying the marked heterogeneity that exists in psychiatric disorders—even within a single diagnostic category. The diagnosis of Attention Deficit Hyperactivity disorder (ADHD) provides a salient example of clinical heterogeneity, as DSM-IV distinguishes between three distinct subtypes, including predominantly hyperactive/impulsive (relatively infrequent), predominantly inattentive, and combined (most frequent among children). While the behavioral literature has long struggled with the challenges of identifying commonalities and differences among the subtypes, imaging studies have generally ignored these distinctions. The second challenge is the development of image processing and analytical methods capable of separating signal from artifact, enabling both the characterization of pathologic processes underlying a given disorder, and detection of their presence in an individual. As a prototypical hyperkinetic disorder, ADHD presents exceptional challenges for fMRI-based research, due to importance of addressing the increased prevalence of motion in this population, which can artificially produce or obscure ADHD-related differences in rs-fcMRI metrics (Power et al., 2012a; Satterthwaite et al., 2012; Van Dijk et al., 2012).

In response to these challenges, here we use an aggregate dataset pooled across five institutions [resulting in quality-controlled rs-fcMRI scans for 455 typically developing children (TDC) and 193 children with ADHD] to provide novel insights into the neural correlates of clinical heterogeneity in ADHD. Specifically, we use support vector machine (SVM)-based multivariate pattern analysis (MVPA) to identify those functional connections in the brain that, collectively, are capable of differentiating the DSM-IV Inattentive and Combined ADHD subtypes (termed presentations in DSM-5) from one another, as well as from typically developing controls (we omit the hyperactive-impulsive subtype due to its relative rarity in the age ranges studied). We believe that such pattern analytic approaches may prove to be advantageous in the examination of ADHD, given the growing consensus that the neural correlates of ADHD are distributed in nature, rather than being explained by abnormalities in any specific connection or region.

In this work we begin with a methodological aim to assess several techniques aimed at controlling for micro-movements in a TDC sample. We focus this analysis on results related to short and long-range functional connectivity, as recent works suggest that findings related to these types of connections can be augmented by micro-movements (Power et al., 2012a; Satterthwaite et al., 2012; Van Dijk et al., 2012). Using the strongest methods from the first aim, we then follow-up this examination to investigate subtype heterogeneity and the predictive capacity of rs-fcMRI in a large group of TDC vs. a large group of children with ADHD.

## Methods

### Participants and measures

Data from Brown University, Beijing Normal University, Kennedy Krieger Institute, New York University Child Study Center, Washington University at St. Louis, and Oregon Health and Science University were aggregated for youth ages 7–14 years. The resulting dataset comprised 455 typically developing control subjects and 193 subjects with a DSM-IV-TR diagnosis of ADHD. A summary of the demographic characteristics for each sample is provided in Table 1. Informed written consent and assent were obtained for all participants, and procedures complied with the Human Investigation Review Board at respective universities. As data for this investigation were aggregated retrospectively (as a large collaborative effort), slightly different ADHD assessment protocols were used across institutions. These procedures are detailed in Appendix text.

**Table 1:** **Sample characteristics**.

	***N***	**Age (mean)**	**% Female**	**FD mean**	**ADHD-I**				**ADHD-C**			
					***N***	**Age (mean)**	**% Female**	**FD mean**	***N***	**Age (mean)**	**% Female**	**FD mean**
Total	455	14.39	50	0.10	80	11.45	27	0.11	112	10.31	19	0.11
Brown	6	12.38	83	0.11	1	14.70	0	0.00	5	11.86	80	0.11
NYU	65	15.54	54	0.10	15	10.54	47	0.12	37	9.95	16	0.11
Beijing Normal	176	13.63	44	0.10	48	12.14	17	0.11	40	11.25	05	0.11
JHU	70	13.78	53	0.09	5	11.27	20	0.08	14	9.77	50	0.09
OHSU	56	13.94	59	0.11	11	9.15	45	0.11	16	8.80	13	0.11
WashU	82	16.09	48	0.11	–	–	–	–	–	–	–	–

*FD mean reflects the mean frame displacement of the remaining frames after censoring (see Methods)*.

### Data acquisition

All participants were scanned on 3.0 Tesla scanners using standard resting-connectivity T2^*^-weighted echo-planar imaging (details for each institution are provided in Appendix text). All imaging data used is publicly available at the Neuroimaging Informatics Tools and Resources Clearinghouse (NITRC), see http://fcon_1000.projects.nitrc.org/indi/adhd200.

### Preprocessing

All functional images were preprocessed in the same manner to reduce artifacts (Miezin et al., 2000). These steps included: (1) removal of a central spike caused by MR signal offset, (2) correction of odd vs. even slice intensity differences attributable to interleaved acquisition without gaps, (3) correction for head movement within and across runs [also see Power et al. (2012a)], and (4) within-run intensity normalization to a whole brain mode value of 1000. Atlas transformation of the functional data was computed for each individual via the MPRAGE scan. Each run then was resampled in atlas space (Talairach and Tournoux, 1988) on an isotropic 3 mm grid, combining movement correction and atlas transformation in one interpolation (Lancaster et al., 1995). All subsequent operations were performed on the atlas-transformed volumetric time series.

Functional connectivity preprocessing followed prior methods (Fox et al., 2005; Fair et al., 2007a,b, 2008, 2009). These steps included: (1) a temporal band-pass filter (0.009 Hz < *f* < 0.08 Hz) and spatial smoothing (6 mm full width at half maximum), (2) regression of the whole brain signal averaged over the whole brain, (3) regression of ventricular signal averaged from ventricular region of interest (ROI), and (4) regression of white matter signal averaged from white matter ROI. Regression of first order derivative terms for the whole brain, ventricular, and white matter signals were also included in the correlation preprocessing. These preprocessing steps are thought to reduce spurious variance unlikely to reflect neuronal activity (Fox and Raichle, 2007).

### Traditional motion parameters and correction

In a typical functional connectivity experiment, motion is addressed by excluding participants with high levels of movement (using various criteria), and then removing movement-related signal via a linear regression of preprocessed data on the 6 motion parameters (i.e., rotation and translation) for remaining participants. In some instances, samples are matched for movement [via parameters such as root mean square (RMS)] (Fair et al., 2007a; Dosenbach et al., 2010). However, these approaches involve potentially problematic assumptions. The first is that the traditional calculations of the 6 motion parameters (which are typically generated relative to a within-run reference frame) are tightly related to abrupt motion-related changes in the BOLD signal. The second is that there is a linear relationship between changes in the BOLD signal and abrupt motion in the scanner. Three recent reports (Power et al., 2012a; Satterthwaite et al., 2012; Van Dijk et al., 2012) suggest that these assumptions are likely incorrect and that traditional motion correction does not adequately control for the changes in signal intensity that accompany abrupt changes in head position.

With this concern in mind, we attempted several motion correction procedures (described below). At the first level of correction (i.e., traditional motion correction), motion was measured relative to a reference frame (in this case, the middle frame of a BOLD run) and quantified using an analysis of head position based on rigid body translation and rotation. This procedure results in the rigid body transform defined by 6 motion parameters (3 translation, 3 rotation) typically generated by most functional MRI software tools. Traditional motion correction procedures in fMRI-based functional connectivity studies, as well as in many task-based fMRI studies, use these 6 parameters as regressors in preprocessing to remove potential motion-related artifact. This step was included in most analyses below.

In addition, in an effort to remove participants with egregious motion, we began our analysis by filtering those subjects with high movement runs based on RMS. The data derived from the 6 motion parameters needed to realign head movement on a frame-by-frame basis were calculated as RMS values for translation and rotation in the x, y, and z planes in millimeters. Total RMS values were calculated on a run-by-run basis for each participant. Participant's BOLD runs with movement exceeding 1.5 mm RMS were removed.

### Frame-to-frame (volume-to-volume) motion parameters

In an effort to examine motion from volume-to-volume, two additional motion parameters were examined. The first, based on framewise displacement (FD), was first introduced by Power et al. (2012a). This variable measures movement of any given frame relative to the previous frame, as opposed to relative to the reference frame (as above). Thus, the method yields a 6 dimensional time series representing frame-to-frame motion, as described by FD_*i*_ = |Δ*d*_*ix*_| + |Δ*d*_*iy*_| + |Δ*d*_*iz*_| + |Δα_*i*_| + |Δβ_*i*_| + |Δγ_*i*_|, where Δ*d*_*ix*_ = *d*_(*i* − 1)*x*_ − *d*_*ix*_, and similarly for the other five rigid body parameters [*d*_*ix*_ *d*_*iy*_ *d*_*iz*_ α_*i*_ β_*i*_ γ_*i*_]. In essence, this formula sums the absolute values of volume-by-volume changes in the six rigid body parameters (Note: Rotational displacements for this method are first converted from degrees to millimeters by calculating surface displacement on a sphere of radius 50 mm, the approximate distance from the cerebral cortex to the center of the head).

A second measure likely to reflect direct BOLD-related deviations secondary to movement is termed DVARS (the RMS of the derivatives of the differentiated timecourses of every brain voxel for each acquired volume), originally described by Smyser et al. (2010). DVARS quantifies volume-to-volume BOLD signal change, thus capturing large deviations produced by phenomena that impact the brain on a global scale—head motion being a major contributor to fluctuations in DVARS. This measure is based on the fact that abrupt head displacement typically manifests as signal loss in echo-planar imaging (Smyser et al., 2010). Thus, a logical measurement sensitive to sudden changes in head position is the whole brain signal change measured by DVARS (Power et al., 2012a). DVARS is computed by aggregating voxel-wise volume-by-volume backwards differences in the BOLD signal described by: DVARS ${\left(\Delta I_{i}\right)}_{t}=\sqrt{\langle {\left[\Delta I_{i}\left(\vec{x}\right)\right]}^{2}\rangle }=\sqrt{\langle {\left[I_{i}\left(\vec{x}\right)-I_{i-1}\left(\vec{x}\right)\right]}^{2}\rangle }$, where $I_{i}(\vec{x})$ is image intensity at locus $\vec{x}$ on frame *i* and angle brackets denote the spatial average over the whole brain. Because frame-to-frame changes in signal intensity related to movement are significantly greater than those caused by neurophysiologic changes in the BOLD signal, this measure provides a natural parameter with which to directly examine the relationship of movement measurements and the BOLD response (Smyser et al., 2010).

### Region of interest selection

We selected 160 regions of interest (ROIs) based on prior work by Dosenbach et al. (2010) (see Figure A7). These regions were selected based on their use to develop maturation indices (fcMI) in a previous study (Dosenbach et al., 2010). This set of regions originated from a series of five meta-analyses, focused on error-processing, default-mode (task-induced deactivations), memory, language, and sensorimotor functions (Dosenbach et al., 2010). The functionally defined ROIs were used as a “best guess” estimate of the underlying functional area architecture across the brain (Fair and Schlaggar, 2008).

Network categorization of each ROI was based on labels designated in a previous report (Dosenbach et al., 2010). This categorization stemmed from a community detection procedure conducted on combined correlation matrices across adult subjects [e.g., see Fair et al. (2009)]. The modularity optimization algorithm of Newman was used (Newman, 2006). The modules (i.e., communities) used to categorize regions had a high quality index (Q) and were the most resistant to perturbation by randomization, measured by variation of information (VOI) (Karrer et al., 2008). For Figure A7, the network assignments were re-examined based on the current adult and child datasets. In this instance, the weight conserving community detection algorithm used was based on the work by Rubinov and Sporns (2011).

### Computation of single subject correlation matrices

SVR analyses in the manuscript were conducted on single subject correlation matrices derived from the above mentioned 160 *a priori* ROIs (10 mm diameter spheres). The resting-state BOLD time series were correlated region by region for each participant across the full length of the resting time series, creating 455 square correlation matrices (160 × 160), one for each subject (Dosenbach et al., 2007; Fair et al., 2007a, 2008, 2009). For those motion correction strategies in which motion covariates were included in the calculation, the resultant matrices for each participant represent partial correlations (after accounting for motion).

### Age relationships

For all statistical comparisons, *r*-values within matrices were first normalized using the Fisher r-to-z transformation. Functional connections of the brain most strongly associated with age were then determined by cross correlating each connection with subject age (in one instance partial correlations were generated using mean movement parameters as a covariate—see below). Connections were corrected with the Benjamini and Hochberg False Discovery Rate (Benjamini et al., 2001). Connection distances for those links that significantly correlated with age were calculated in terms of Euclidean distance (i.e., vector distance) between the center of mass coordinates of each region. These values were then separated for those connections that got stronger with age or weaker with age. *Post-hoc* analyses for these same connections using one of the selected movement-related procedures below (i.e., Procedure 8) was conducted for Figure A2 (site by site comparisons).

### Support vector-based multivariate pattern analysis

In the present study SVM and support vector regression (SVR)-based MVPA were used to make predictions about brain maturity and disease status in individual subjects (e.g., Norman et al., 2006; Dosenbach et al., 2010; Rizk-Jackson et al., 2011). The approach used in the present analysis was similar to that used in a previous report (Dosenbach et al., 2010).

SVM-based MVPA is a supervised classification algorithm rooted in statistical learning theory. Conceptually, input vectors are mapped to a higher dimensional feature space using special non-linear functions called kernels. Classification is performed by constructing a hyperplane in the feature space that optimally discriminates between two classes of the training data by maximizing the margin between two data clusters.

Given a training set of the form (**x**_*i*_, *y*_*i*_) where the vectors **x**_*i*_ are data points and *y*_*i*_ are the class labels, the SVMs require the solution to the following optimization problem:

$$\underset{w,b,\xi }{\min}\frac{1}{2}w^{T}w+C\sum_{i=i}^{n}\xi_{i}$$

subject to *y*_*i*_(***w*** × ***x***_*i*_ + *b*) ≥ 1 − ξ_*i*_ and ξ_*i*_ ≥ 0 where ξ_*i*_ are the slack variables, measuring the degree of a data point's misclassification, ***w*** are the weights defining the hyperplane and *C* > 0 is the penalty parameter of the error term. The resultant decision function implemented by SVM can be written as:

$$f\left(x\right)=sign\left(\sum_{i=1}^{n}y_{i}\alpha_{i}K\left(x,x_{i}\right)+b\right)$$

where *K*(*x*_*i*_, *x*_*j*_) is the kernel function. In our work, we use the radial basis kernel given by:

$$K\left(x_{i},x_{j}\right)=exp\left(-\frac{{\left\|x_{i}-x_{j}\right\|}^{2}}{2\sigma^{2}}\right)\text{​​},$$

SVMs are inherently two-class classifiers. Multiclass SVM aims to handle the *K*-class pattern classification problem by reducing a single multiclass problem into multiple binary classification problems. The most common method for such reduction is to build a set of *one-vs.-rest* binary classifiers that distinguish one of the classes from the rest. Another strategy is to build a set of *one-vs.-one* classifiers that distinguish between every pair of classes. For the *one-vs.-one* approach, classification is done by max-wins voting strategy that chooses the class that is selected by the most classifiers. For the *one-vs.-rest* case (used in this work), classification of new instances is done by a winner-takes-all strategy, in which the classifier with the highest output function assigns the class. SVM classifications used a soft margin *C* = 1, and a radial basis function with σ = 2. In brief, for all SVR classifications we used epsilon-insensitive SVRs. Parameters were set with *C* = Infinity, epsilon = 0.00001 with σ = 2 [as in Dosenbach et al. (2010)]. We use Spider (http://people.kyb.tuebingen.mpg.de/spider/main.html), an object orientated environment for machine learning in Matlab (MATLAB 7.1.0, The Mathworks, Natick, MA), for generating the SVM models.

**Figure A1:**
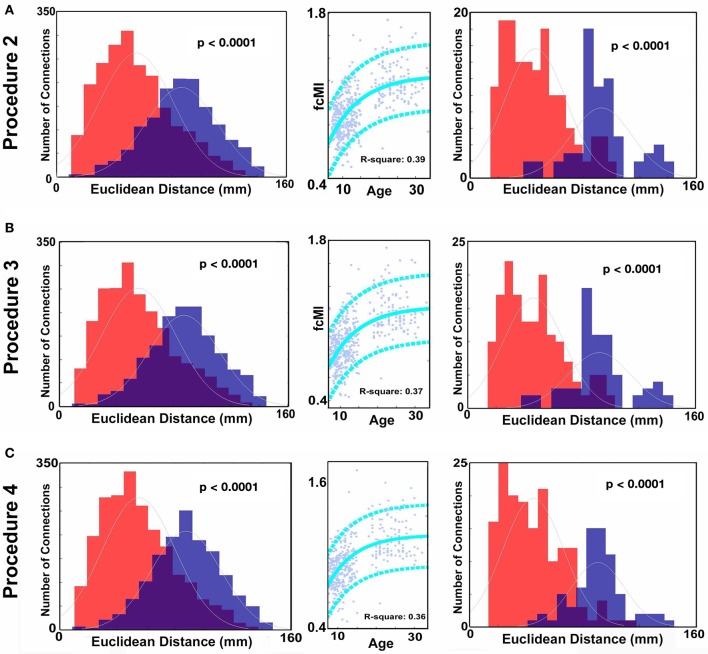
**For procedures 2 (A), 3 (B), and 4 (C) (as in Figure 2) on left histograms of Euclidean distance for functional connections that get stronger with age and those that get weaker with age (FDR corrected).** As in Figure 3 (middle) SVR-based MVPA brain maturation curves and on right Euclidean distance of consensus features that grow up with age vs. those that grow down with age.

The brain functional connectivity maturity index (fcMI) for TDC (Figures 3 and A1) was based on the top 300 connections that correlated (and passed FDR correction) in each round of leave-one-out-cross-validation (LOOCV). Calculating fcMI by LOOCV involves removing a single subject as a test sample, then using the remaining data for feature selection and as the training set for the SVR predictor. This procedure is then repeated until each subject is used once as the test case [i.e., univariate feature-filtering, see De Martino et al. (2008); Pereira et al. (2009)].

We also conducted an SVM analysis using features (i.e., connections) that most strongly differentiated ADHD subjects of each of two subtypes [i.e., primary inattentive type (ADHD-I) and combined type (ADHD-C)] from each other and/or control subjects (2-group classification: *t*-test; 3-group classification: ANOVA). For this procedure, we ran the SVM using LOOCV on all subjects conducted on the top 150 features that differentiated each subtype and controls (via the comparisons as noted above). These analyses are presented in Figure 8 and Figures A4 and A5. The number of features used in this case was reduced because of our reduced sample size for the comparisons.

**Figure 1:**
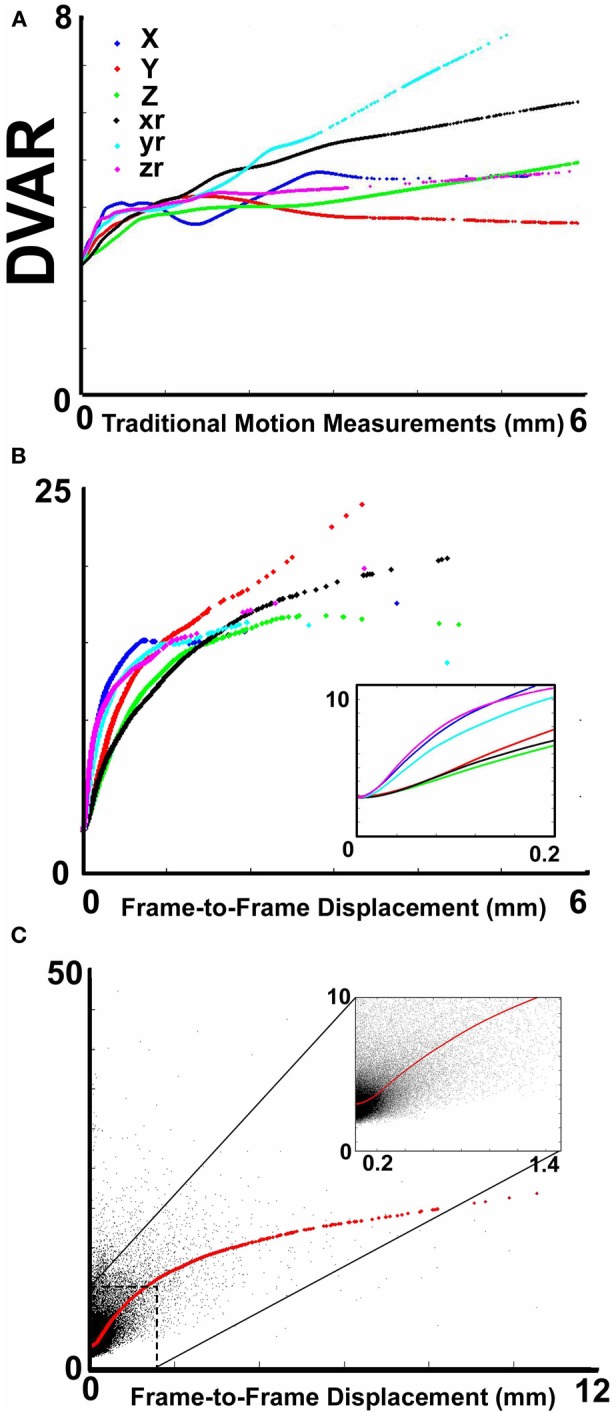
**The relationship between movement parameters and variations in echo-planar imaging. (A)** LOWESS curves of six traditional movement measurements, which align any given frame within a run to a reference frame within that run, relative to the derivation in BOLD signal (i.e., DVARS) from volume to volume (or frame to frame). These traditional movement measurements show a non-linear relationship with changes in the BOLD signal. **(B)** The same plot as in **(A)**, using 6 frame-to-frame motion measurements as opposed to traditional movement parameters. **(C)** FD, which combines all 6 directions in **(B)**, as a function of DVARS. There is a stronger relationship of DVARS and the FD measurements, compared to traditional motion estimates shown in **(A)**.

For the typically developing population, the SVR and univariate age relationships were conducted after 10 potential motion correction procedures (Table 2). Procedure 1 used traditional motion correction, which included regression of the BOLD data on 6 rigid body motion parameters (based on the middle reference frame) during preprocessing. Procedure 2 added an additional step that included the six rigid body frame-to-frame parameters, [Δ*d*_*ix*_ Δ*d*_*iy*_ Δ*d*_*iz*_ Δα_*i*_ Δβ_*i*_ Δγ_*i*_], as covariates to generate partial correlation matrices (160 × 160) for each participant. Procedure 3 was similar to Procedure 2, but used FD_*i*_ as the covariate. Procedure 4 was the same as Procedure 1, but replaced the traditional preprocessing motion regressors (i.e., Procedure 1) with the frame-to-frame measures (i.e., [Δ*d*_*ix*_ Δ*d*_*iy*_ Δ*d*_*iz*_ Δα_*i*_ Δβ_*i*_ Δγ_*i*_]). Procedure 5 utilized a group-level correction, similar in nature to what has been proposed by Van Dijk et al. (2012). In this instance, mean FD for each participant is used as a covariate when cross correlating each connection across subject age. Procedure 6 involves a movement “matching” procedure (analogous to matching a parameter between groups) whereby mean FD is used to remove subjects until there is no relationship between mean FD and age (Figure 5). Procedure 7 is a method (i.e., “scrubbing” or volume censoring) first proposed by Power et al. (2012a). In this instance, frames are removed based on the magnitude of FD_*i*_. In the original publication frames were removed based on having FD_*i*_ > 0.5 mm and a DVARS level greater than 5. Here we apply a more stringent criterion based on findings in Figure 1, and remove frames prior to creating the 160 × 160 correlation matrix for each subject based on an FD_*i*_ > 0.2 mm. To account for potential temporal blurring of the motion-related artifact during the bandpass filter, 2 frames after the censored frame, and one frame before are also removed (Power et al., 2012a). Procedure 8 is an extension of procedure 6 and combines matching with frame removal. Here additional participants are removed to assure there is no relationship of mean FD and age for the *remaining* frames after applying frame removal based on FD_*i*_ > 0.2. Procedure 9 was identical to Procedure 8, but DVARS_*i*_ is used to remove frames instead of FD_*i*_. Here DVARS_*i*_ > 4 was used to remove frames likely associated with excess movement. In addition, subjects were removed so there was no relationship between mean DVARS and age (see Figure 5). Procedure 10 aims to utilize the censoring techniques described above; however, it does so only to generate the effect of movement on the correlation coefficients in each subject. It then utilizes this information to correct the original values. In this sense r-values are corrected via censoring, but without requiring actual data removal for the correlation estimates. For the procedure, connectivity matrices are first generated from the original and censored time series (i.e., Procedure 7) for each subject. Next, within each participant, delta r is calculated for all of the connections (i.e., the difference between the scrubbed and the original connectivity data is calculated for each individual subject). A polynomial curve is fitted to the relationship between delta R and distance between the ROIs (see Figure A8). The general form of a polynomial equation is shown below.

$$y=p_{1}x+p_{2}x^{2}+p_{3}x^{3}+\cdots +p_{n}x^{n}+p_{n\;+\;1}$$

**Table 2:** **Brief description of each motion correction procedure**.

**Procedure**	**Method**
1	Traditional motion correction: regression of BOLD data with six rigid motion parameter in pre-processing
2	The six rigid body frame to frame parameter were used as a covariates to generate partial correlation matrices
3	Same as Procedure 2 but uses FD as a covariate
4	Same as Procedure 1 but regressed the BOLD data with six rigid body *frame to frame* parameters
5	Group level correction: mean FD is covaried while cross-correlating connections across subject age
6	Movement matching: removed subjects to make sure there was no relation between mean FD and age
7	Frames having FD > 0.2 mm were removed (scrubbing). To account for temporal blurring one frame before and two after were also removed
8	This is combining Procedure 7 and Procedure 6 i.e., frame censoring and movement matching was done
9	Same as Procedure 8. Instead of FD > 0.2, frames were removed based on DVAR > 4, then the movement matching was done with mean DVAR and age
10	Utilizes a polynomial generated for each subject based on information obtained from Procedure 7 to correct for the effect of movement

As shown in the Figure A8, for a given subject, the red curve shows the trend of delta r and the blue curve shows the curve fitted to delta r (Note: in the figures we also include LOWESS curves of the data to visualize how the polynomial fits the data). The method is applied on each subject individually such that every subject will have an equation that fits their respective curve. This equation is then used to regress the effect of movement on the original r-values. Censoring the frames as in Procedure 7 after applying this procedure reveals no effect of movement (Figure A8).

### Visualizing regional brain contributions

Regional brain contributions associated with age or ADHD status were not equally distributed throughout the cortex. Some regions had many connections that strongly correlated with age (or differentiated TDC from ADHD subjects), while others had few or none. As such, we represented this phenomenon with a parameter termed “Node Strength” by scaling the diameter of each node by the summed z-scores of each of its connections identified as a connection of interest (COI) in previous analyses. Node strength is simply the sum of all of the weights (in this case z-scores) attached to a specific node [e.g., see Hagmann et al. (2010)]. Node strength was calculated for the consensus features (those features present in each round of the LOOCV procedures above) that most strongly correlated with age, as well as for the strongest feature set found in the comparisons with regard to the ADHD subtypes. If an ROI did not have any COIs that correlated with age or differentiated groups based on our criterion, it was assigned a Node Strength of 0. Nodes with many and/or strong connections that correlated with age or differentiated groups have a high node strength. Raw values for all Node Strengths are presented in Table A1. Surfaced-based mapping of nodes and vectors were conducted using caret software (Van Essen et al., 2001; Van Essen, 2005).

## Results

Given prior demonstrations of increased micro-movements in pediatric samples and the potential for motion artifact to contribute to estimates of functional connectivity (Power et al., 2012a; Satterthwaite et al., 2012; Van Dijk et al., 2012), we first provide a comprehensive examination of micro-movements in the ADHD-200 sample, as well as the impact of various correction strategies. Then, we report unique findings from our ADHD-C and ADHD-I samples, after controlling for movement using the most conservative procedure.

### Section 1: characterizing and accounting for micro-movements in the ADHD-200 sample

We first examined the relationship between traditional movement measures and abrupt changes in the BOLD signal as measured across the whole brain. Traditional movement measurements relate to the six directional adjustments (i.e., rotation and translation) required to align any given frame within a BOLD fMRI run to a reference frame within that run. As noted in methods, this reference frame can be any frame (e.g., in AFNI), but often is the middle frame of a run (e.g., in FSL). Our measurement for changes in the whole brain signal is termed DVARS. Figure 1A clearly illustrates that there is a non-linear relationship between all six traditional motion parameters and changes in the BOLD signal. These findings violate the assumptions inherent to several traditional preprocessing movement correction procedures (e.g., linear regression), for both traditional task and connectivity-based fMRI analyses.

Importantly, however, while the relationship of frame-to-frame motion parameters (i.e., FD) and changes in the whole brain signal is positive, there is still a non-linear relationship (Figure 1B). These data suggest that simply replacing traditional movement parameters with frame-to-frame motion parameters in traditional preprocessing steps (i.e., linear regression) will assist, but likely not fully account for potential movement artifact. This presumption was confirmed in the following analyses (also see Figure A1).

#### Traditional motion correction in a typically developing sample (Procedure 1)

We begin this section by first identifying the functional connections that most strongly associated with age in TDC, when only traditional motion correction procedures were used. Again, for each subject, we estimated the Pearson *r* between the resting BOLD time-series computed for 160 *a priori* defined brain ROIs (Dosenbach et al., 2010). After Fisher r-to-z transformation, this step created 455 square *r*-matrices (160 × 160)—containing 12,720 pair-wise connections for each participant. Two analyses were applied to examine age relationships. The first was a basic cross-correlation to examine how each pair-wise connection relates with age. The second was the use of SVR-based MVPA (Norman et al., 2006; Rizk-Jackson et al., 2011) to verify the ability of rs-fcMRI to predict brain maturity for TDC. Chronological age served as our training measure, while predicted age was our measure of functional maturity. We calculated the fcMI by setting the model fit (Von Bertalanffy's growth curve model [a•(1 − e^−*bx*^)]) of predicted age for the oldest subjects equal to one. Predictions for each TDC subject were based on the top 300 connections in each round of LOOCV.

**Figure 2:**
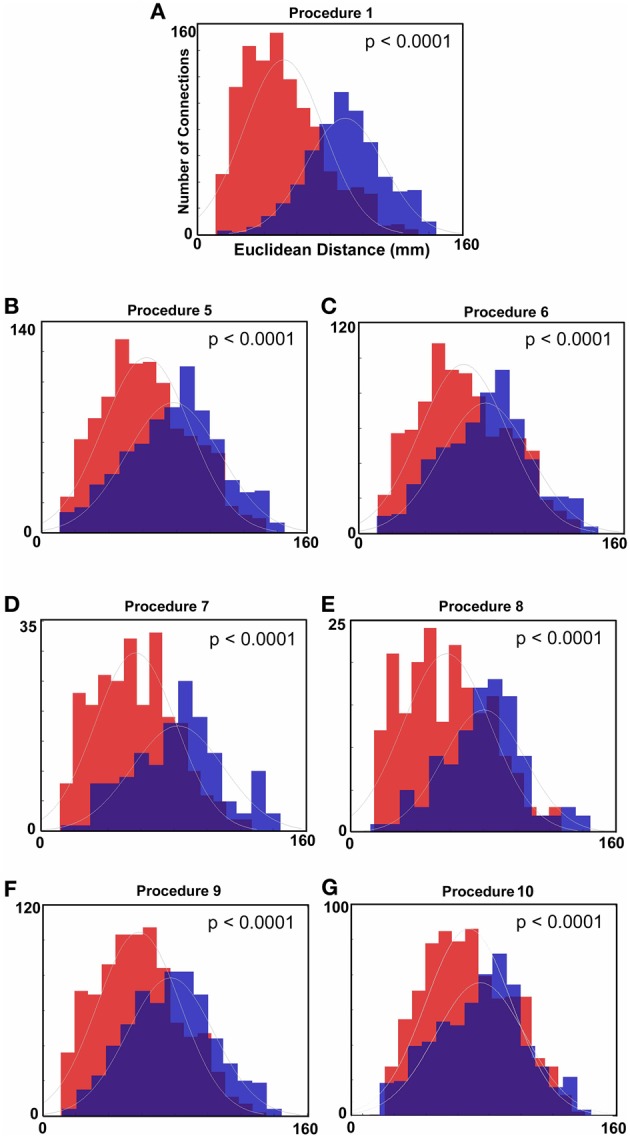
**Histograms of Euclidean distance for functional connections that get stronger with age and those that get weaker with age (FDR corrected).** Red colors denote the distribution with regard to distance of those connections that get weaker with age. Blue colors represent the distribution for those connections that get stronger with age. **(A)** Distance measurements after traditional motion correction (Procedure 1), **(B)** Procedure 5, **(C)** Procedure 6, **(D)** Procedure 7, **(E)** Procedure 8, **(F)** Procedure 9, and **(G)** Procedure 10. Table 2 provides a short summary of each of these procedures. Each motion correction procedure reduced the mean difference in distance between those connections that get stronger relative to those that get weaker with age. Also see Figure A1 for procedures 2–4.

**Figure 3:**
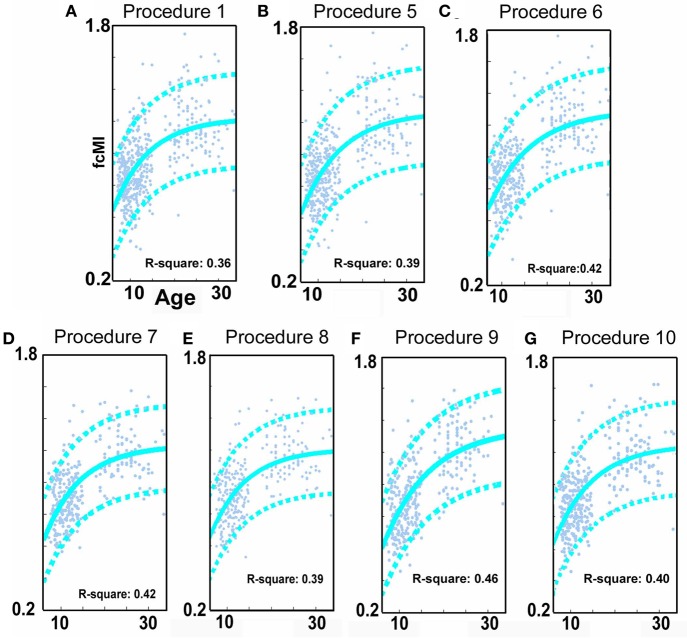
**SVR-based MVPA brain maturation curves**. SVR-based MVPA brain maturation curves showing individual age predictions for TDC (blue) after seven motion correction procedures. Chronological age is presented on the x-axis and fcMI on the y-axis. The Von Bertalanffy's growth equation shows a reasonable fit for these data (with 90% prediction limits shown with dashed lines). **(A)** Procedure 1 (traditional motion correction), **(B)** Procedure 5, **(C)** Procedure 6, **(D)** Procedure 7, **(E)** Procedure 8, **(F)** Procedure 9, and **(G)** Procedure 10. Also, see Methods and Table 2 for short description of each procedure.

**Figure 4:**
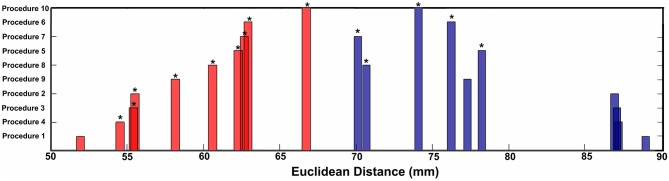
**Mean Euclidean distance for short-range and long-range connection changes over age for each procedure.** Asterisks represent significant differences in distance relative to Procedure 1 (traditional motion correction).

Consistent with prior reports (Fair et al., 2007a, 2009; Kelly et al., 2009; Supekar et al., 2009; Dosenbach et al., 2010), we found that connections that became stronger with increasing age, or “grew-up,” tended to link distant regions (i.e., long-range connections). Connections that became weaker with increasing age, or “grew-down,” were primarily found between proximal regions (i.e., short-range connections) (see Figures 2 and 4; *p* < 0.0001). We also saw robust age-predictions for the TDC group. Von Bertalanffy's growth curve model ([a•(1 − e^−*bx*^)]) provided a significant fit for predicting brain maturity (Figure 3A) (*R*^2^ = 0.36). These results served as our baseline.

#### Using frame-to-frame measures as covariates when correlating time series (Procedures 2–4)

We next examined the same age relationships using the additional (or alternative) procedures focused on micro-movement correction. Here, in addition to performing the traditional correction for motion (Procedure 1), we computed partial correlation estimates between the BOLD time series of each of the 160 ROIs using either (1) Frame-to-frame displacement (FD_*i*_) as a covariate, or (2) the frame-to-frame 6 motion parameters as unique covariates (i.e., [Δ*d*_*ix*_ Δ*d*_*iy*_ Δ*d*_*iz*_ Δα_*i*_ Δβ_*i*_ Δγ_*i*_]). This step created 455 square *partial* correlation matrices for each method. Again, after Fisher r-to-z transformation, each pair-wise connection was cross-correlated with age. As can be observed in Figures A1 and 4, the distinction between short- and long-range connections in terms of their relationships with age was only modestly altered by these additional correction procedures, while the ability to make age predictions was maintained. Similar findings were observed when we replaced the traditional movement regressors in the preprocessing with regressors based on FD_*i*_ (Figures A1 and 4). The limited effect of these procedures on the short-long range distinctions is likely secondary to the non-linear relationships between changes in BOLD and movement (Figure 1).

#### Using mean FD as a covariate with age (Procedure 5)

Similar to prior work by Van Dijk et al. (2010), we next examined the effect of including mean FD_*i*_ across all frames for each subject as a group-level covariate in the calculation of the correlation between functional connectivity and age. As previously shown, this method substantially attenuates the distinction between long- and short-range connections in terms of their relationships with age (Van Dijk et al., 2012) (see Figures 2 and 4). The difference in the mean distance of short-range connections using this method relative to those obtained using Procedure 1 (i.e., traditional correction) was highly significant (*p* < 0.0001). The difference in mean distance of long-range connections using this method relative to Procedure 1 was also highly significant (*p* < 0.0001) (Figure 4). Interestingly, while the short-long range effect was reduced, the ability to make age predictions in the data rose slightly from the original estimate (i.e. Procedure 1) with the movement correction procedures (*R*^2^ = 0.39).

#### Subject “matching” based on mean FD_i_ (Procedure 6)

**Figure 5:**
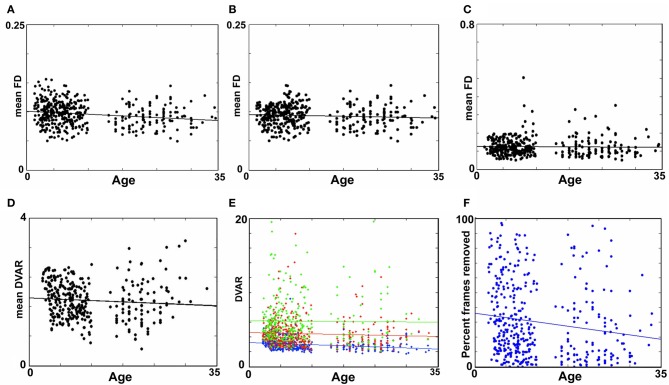
**Age relationships of movement-related measurements. (A)** Mean FD as a function of age for Procedure 7 (i.e., mean FD for the remaining frames after removal). There is a small, but significant relationship with mean FD and age (*p* < 0.01). **(B)** Procedure 8 after matching shows that the relationship found in **(A)** is no longer significant (*p* = 0.13). **(C)** Mean FD as a function of age for Procedure 6 (matching only without frame removal). Again, here there is no relationship with mean FD and age (*p* = 0.62). **(D)** A similar figure as in **(B)**, for the DVAR based frame removal (i.e., Procedure 9). As with **(B)** and **(C)**, there is no age relationship with the DVAR measurement (*p* = 0.13). **(E)** Using mean FD as a covariate (i.e., Procedure 5) with age assumes that movement affects the BOLD signal similarly for any age group. Thus, for all subjects, data frames were split into low (FD = 0 − 0.2), medium (FD = 0.2 − 0.4), and high (FD = 0.4 − 0.6) movement for each participant. The age relationship with our whole brain BOLD measurement, DVARS, was then examined for each bin. While there is a limited age relationship in the lowest movements overall the data suggest movement affects the BOLD signal similarly across the age range studied here (blue *p* < 0.01, red *p* = 0.25, green *p* = 0.88). **(F)** The percentage of frames removed across age for Procedure 8 (i.e., FD frame censoring after matching), as expected shows a slightly higher rate of frame removal for the younger participants.

We next employed a method that excluded subjects on the basis of their mean FD until there was no relationship between the measure and age (*p* = 0.63) as shown in Figure 5C. Because there appears to be no strongly discernible relationships between the effect of movement on the BOLD signal and age (i.e., the effect of FD on DVARS is similar for all ages—Figure 5), we expected this analysis to yield similar results as using the measure as a covariate with regard to our outcome measure (albeit weaker overall significance levels due to the decrease in sample size). For this analysis we removed participants (*N* = 76) until there was no relationship between mean FD_*i*_ and age (see Figure 5C). We then re-ran the age cross-correlation. This analysis also attenuated the distinction between short- and long-range connections in terms of their relationships with age (Figures 2 and 4). The difference in mean distance of short-range connections using this method relative to no “micro-movement” motion correction (i.e., Procedure 1) was also highly significant (*p* < 0.0001). The difference in mean distance of long-range connections using this method relative to no “micro-movement” motion correction was significant, as well (*p* < 0.0001) (Figure 4). The ability to make age predictions increased (*R*^2^ = 0.42) (Figure 3).

#### Volume censoring (Procedure 7)

Another method recently employed to correct for motion simply censors or removes volumes or frames corresponding to excessive movement (Power et al., 2012a). We used FD_*i*_ > 0.2 mm displacement as our threshold for removal of frames. The basis of this threshold was rooted in Figure 1, which highlights a knee in the LOWESS curve at, or just below, ~0.2 mm FD. Updated recommendations by (Power et al., 2012b) independently established the same threshold. While age-relationships were not completely removed, we once again see a reduction in the distinction noted between short- and long-range connectivity (Figures 2 and 4). The ability to make age predictions based on SVR-based MVPA was similar to Procedures 5 and 6 (*R*^2^ = 0.42).

**Figure 6:**
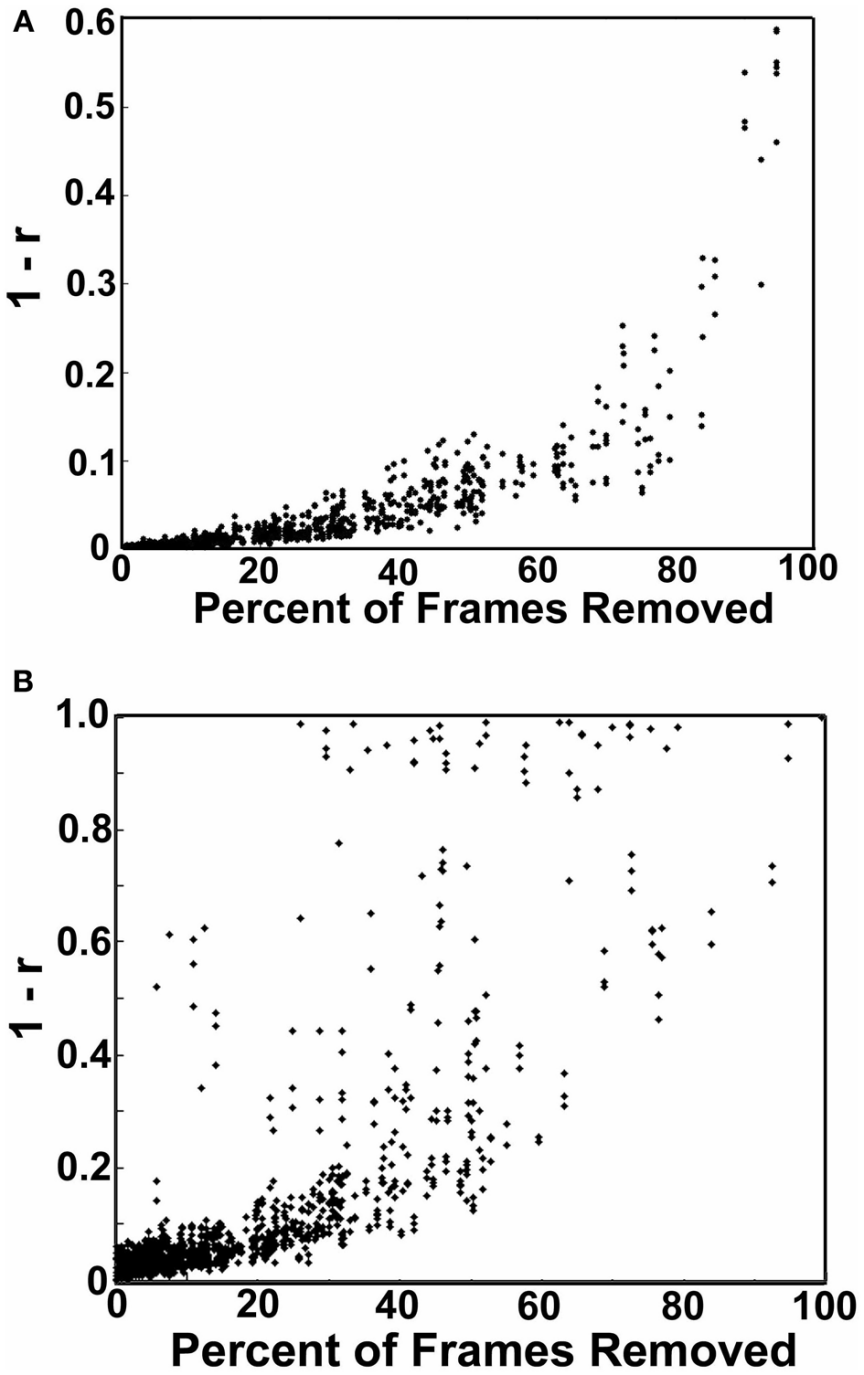
**Simulations showing the effect of frame removal on the correlation structure of a subject matrix.** The simulation began by selected 3 participants with little to no movement based on the 0.2 mm FD threshold (i.e., 0–1% of frames) from each datasets used here (i.e., each institution). For each of these subjects we then calculated their correlation matrix across the 160 ROIs, which served as our baseline. We then iteratively removed frames from the time courses of these subjects based on the frames that did not pass the 0.2 mm displacement criteria for each of the other subjects in their cohort (based on FD_*i*_). In each instance, we compared the newly generated matrices to the baseline matrices of the three subjects that had no frames removed. In **(A)** we show the distance plots (i.e., 1–r) of the original, baseline matrices relative to the simulated matrices as a function of the percentage of frames removed. The distance is low when few frames are removed; however, the tight relationship degrades as the fraction of frames removed approaches 1. In **(B)** we show the same procedure except in this instance missing frame are imputed via a cubic spline interpolation. Substantially more noise is added by interpolation.

One concern of this approach is the consequence of missing, or removing, values in time-series analysis. We attempted to examine this influence with a simulation. The simulation began by selecting 3 participants with little to no movement based on the 0.2 mm threshold (i.e., 0–1% of frames above the threshold) from each dataset (i.e., each institution). For each of these subjects we than calculated their correlation matrix across the 160 ROIs, which served as our baseline (i.e., the true correlation structure). We then iteratively removed frames from the time courses of these subjects based on the frames that did not pass the 0.2 mm displacement criteria for each of the other subjects in their cohort (based on FD_*i*_). In other words if *subject 1* had 30 frames that did not pass criteria, we would remove those same 30 frames from the 3 participants with little to no movement and then recalculate the correlation matrices for those 3 participants. The procedure was next repeated for subject 2, and so on. In each instance, we were able to compare the newly generated matrices to the baseline matrices of the 3 subjects that had no frames removed. Thus, the simulation gave us a pure reference by which we could systematically quantify the effect of removing a given percentage of frames on the overall correlation matrix's structure. Importantly, the number, percentage, and frequency of frames removed for each iteration conformed directly to real data. Figure 6 shows the distance plots (i.e., 1–r) of the original, baseline, matrices relative to the simulated matrices as a function of the percentage of frames removed. As can be seen, the distance is low when few frames are removed; however, the tight relationship degrades as the fraction of frames removed approaches 1. We therefore re-ran our analysis for Procedure 7 while including only those subjects with <60% of their frames removed. Results were largely convergent (see Figure A6).

We also attempted to examine the possibility of replacing missing frames via a common spline interpolation in our simulation [similar in concept to Carp (2011)]. As can be seen in Figure 6, imputing missing frames using cubic spline interpolation added significantly more noise than simply removing the volumes of high movement. As such, we did not attempt to interpolate frames for any of the following analyses.

#### Volume censoring with subject “matching” using mean FD_i_ (Procedure 8)

**Figure A2:**
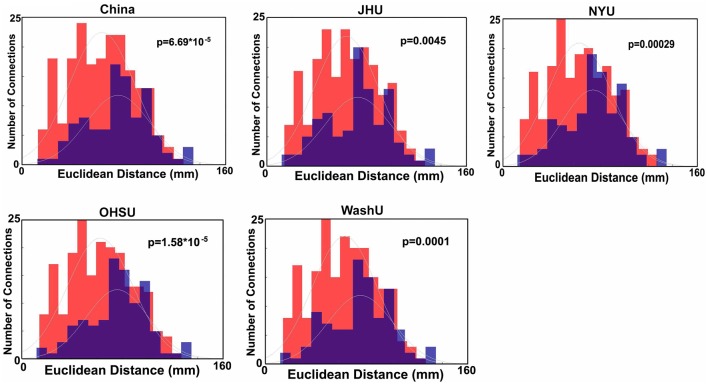
**Site-by-site histograms of Euclidean distance for functional connections identified in the whole group analysis that get stronger with age and those that get weaker with age (FDR corrected) using procedure 8**.

Importantly, despite the removal of motion related frames exceeding a threshold of 0.2 mm displacement, age, and mean FD continued to be associated, *p* = 0.0001 (Figure 5A). This observation is likely due to a small increase in movement in younger subjects for the remaining frames and what can be observed as a small relationship of frame-to-frame changes in BOLD (i.e., DVARS) and FD even under this 0.2 mm cutoff (Figure 1). As such, we repeated the age correlation after combining this censoring approach (Procedure 7) with mean FD matching (Procedure 6—as in Figure 5B). Thus, we repeated the analysis after removing participants (*N* = 142) until the there was no relationship of mean FD_*i*_ (for the remaining frames) and age (*p* = 0.13). This combined method also reduces the short- and long-range relationships (Figures 2 and 4), but shows a slight decline in the ability to make age classifications in individuals (*R*^2^ = 0.34). [Note: findings were consistent across individual sites (Figure A2)].

#### Volume censoring with subject matching using mean DVARS (Procedure 9)

Here we examine the utility of using the DVARS measure itself to remove frames. In this instance, instead of removing frames based on FD_*i*_, we removed frames based on changes in DVARS_*i*_ (DVARS_*i*_ > 4). We also removed participants (*N* = 130) to assure no relationship with DVARS and age for the remaining frames. This analysis is shown in Figures 2, 4, and 5D. Interestingly, while the short- and long-range distinctions in terms of their relationship with age were also significantly reduced as with other methods, the ability to make age predictions was the highest of all of the methods thus far (*R*^2^ = 0.46) (Figure 3).

#### R-value correction using polynomial regression based on volume censoring (Procedure 10)

Finally, we examine a method that applies an r-value calibration by utilizing a polynomial regression based on the correction induced by data scrubbing, as outlined in the methods. Importantly, this particular method is not completely devoid of the issues noted above for data censoring. To obtain a valid estimate of the true polynomial, the method still requires a sensible number of remaining frames. As such, we ran the analysis on subjects with at least 40% of their frames remaining after censoring. The analysis is shown in Figures 2, 4, and 5. As with the other procedures, the short- and long-range distinctions in terms of age were significantly reduced, yet the ability to make age predictions remained (*R*^2^ = 0.40; Figure 3).

**Figure 7:**
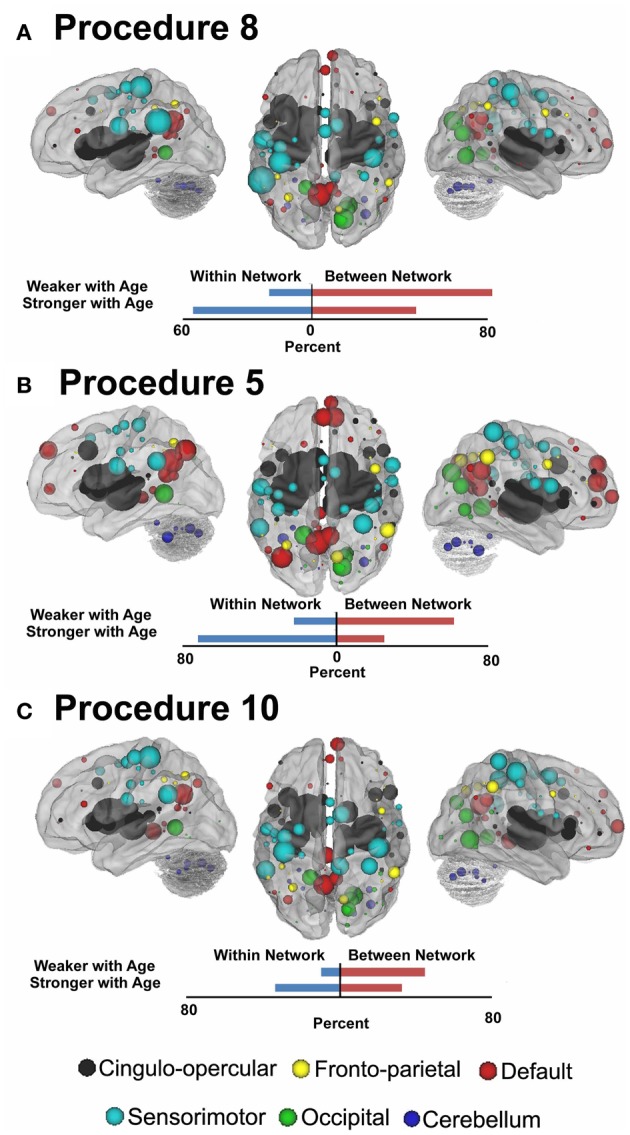
**Node strength for the consensus features that best predict age for Procedure 8 in (A), Procedure 5 in (B), and Procedure 10 in (C).** Within and between network comparisons are also presented for those connections that get stronger with age vs. those that get weaker with age.

**Figure A3:**
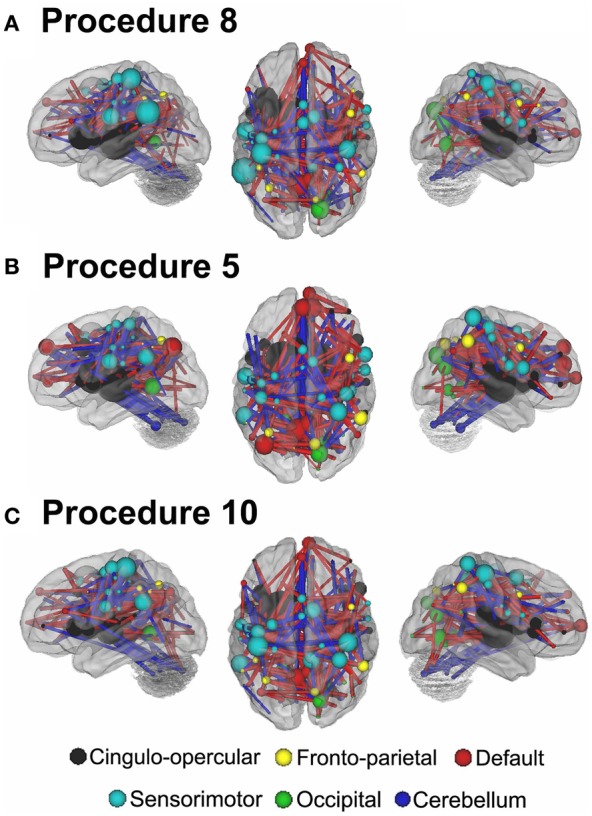
**As with Figure 7, Node strength for the consensus features that best predict age for Procedure 8 in (A), Procedure 5 in (B), and Procedure 10 (C).** Included are the vectors, which are used to calculate node strength.

In Figure 7 (also see Figure A3), for this procedure along with procedures 5 and 8, we use node strength, a commonly employed graph-theory metric (see Methods), to reveal the nodes that are most strongly predicative of age. The figure shows a distributed pattern of connectivity with several subcortical structures being highly predictive. Connections that tended to get stronger with age appeared to be within networks, and connections that got weaker with age were between networks. The findings were qualitatively similar across the motion correction methods.

### Section 2: prediction of diagnostic status

Consistent with prior work (Dosenbach et al., 2010), we followed our movement analyses with an effort to determine if rs-fcMRI in combination with SVM-based MVPA is capable of predicting ADHD status in children with ADHD-C or ADHD-I. Predictions for each subject were based on the top 150 connections (features) that differentiated each subtype from controls in each round of LOOCV. To avoid biasing our classifier group, subsample size was matched (see Table 3). Consensus features, or those connections present across all of the LOOCV samples are provided in Figure A5. In addition, node strength was used to further examine the functional neurobiology that differentiated the two ADHD subtypes from controls. Raw absolute values of node strength are provided in Table A1.

**Table 3:** **Subject characteristics for ADHD and control comparisons**.

		***N***	**Age (mean)**	**% Female**	**IQ**	**FD mean (pre-censor)**	**FD mean (post-censor)**
Total	TDC	52	11.12	0.48	115.83	0.12	0.10
	ADHD-I	52	11.63	0.23	108.27	0.14	0.10
	ADHD-C	52	10.98	0.17	109.04	0.13	0.10

For prediction analyses and subtype comparisons we used what we saw as the three most conservative movement correction approaches, Procedures 5, 8, and 10. These are not necessarily “the best” procedures, as performance varied based on whether we examined short- or long-range connections. Importantly, these analyses were careful to consider potentially confounding variables (site, gender, and IQ) by controlling for them using multiple regression analysis. That is, these variable connections were regressed from functional connectivity values across ROIs connections of all subjects. When adjusting for the confounding variables, our regression equation is:

$$y=\beta_{0}+\beta_{1}x_{1}+\beta_{2}x_{2}+\beta_{3}x_{3}+\beta_{4}x_{4}+\varepsilon ,$$

where *y* represents the vector of connectivity values, β_0_ is the intercept, ε is the vector of residuals, β_1_ to β_4_ represent regression coefficients corresponding to independent or explanatory variables: x_1_—ADHD Diagnosis, *x*_2_—Site, *x*_3_—Gender, and *x*_4_—IQ. The unknown β_*i*_'s that measure the sample relationship between *y* and *x*_*i*_ after all other variables have been partialled out, are obtained using ordinary least squares (OLS) estimation. Using the OLS estimates of β_*i*_, functional connectivity values adjusted for confounding variables are given by:

$$y=\beta_{0}+\beta_{1}x_{1}+\varepsilon .$$

Using procedure 5, our predictions for ADHD-C relative to controls showed 77.0% accuracy (75% sensitivity and 76.9% specificity), while our predictions for ADHD-I relative to controls showed 80.8% accuracy (78.9% sensitivity and 82.7% specificity). Using procedure 8, our predictions for ADHD-C relative to controls showed 63.4% accuracy (61.5% sensitivity, 65.4% specificity), while our predictions for ADHD-I relative to controls showed 78.8 % accuracy (75.0% sensitivity, 82.7% specificity). Last, using procedure 10, our predictions for ADHD-C relative to controls showed 71.2% accuracy (73.1% sensitivity, 69.2% specificity), while our predictions for ADHD-I relative to controls showed 82.7% accuracy (78.9% sensitivity, 86.5% specificity).

We also attempted a 3-group classification for which chance is 33%. For procedure 5, the 3-group classifier revealed results that were highly significant (overall 69.2% accuracy; TDC = 67.3%, ADHD-C = 71.1%, ADHD-I = 69.2%). For procedure 8, the results were also promising albeit reduced (overall 56.4%; TDC = 59.6%; ADHD-C = 44.2%; ADHD-I = 65.4%). For procedure 10 the findings again revealed largely consistent strong findings (overall 68.6%; TDC = 71.2%, ADHD-C = 63.5%, ADHD-I = 71.2%).

#### Characterizing the neural correlates of ADHD subtypes

To examine the potential neurobiological differences in subjects with inattentive and hyperactive symptoms (i.e., combined type), and those with inattentive symptoms only (i.e., predominantly inattentive type), we examined the features that most strongly differentiated the groups for each of the movement correction procedures. That is, we calculated *node strength* for each group comparison as described in methods.

**Figure 8:**
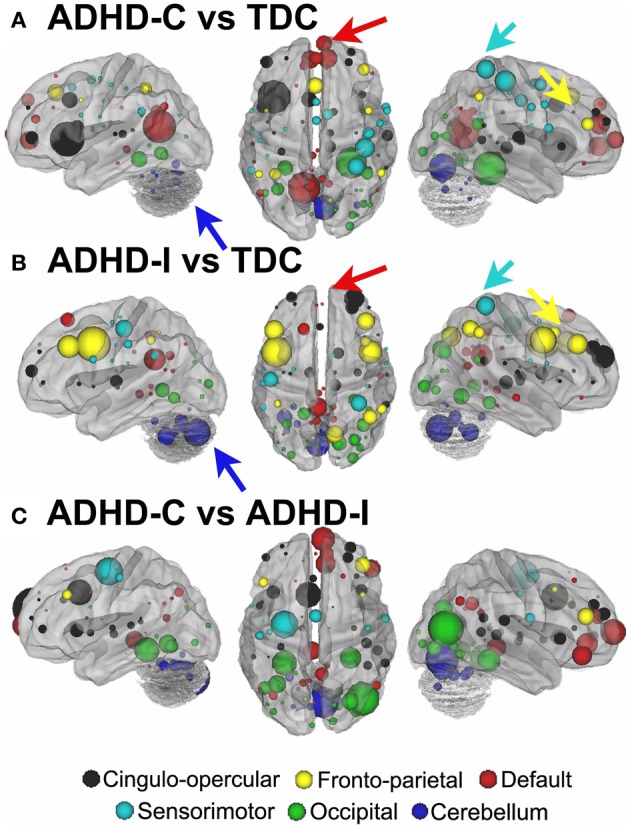
***Post-hoc* comparisons on the consensus features from the 2-group classification for Procedure 10 (see SI Figure 4 for Procedures 5 and 8).** The two most common subtypes of ADHD (ADHD-I and ADHD-C) show distributed patterns of atypical connectivity relative to TDC, as measured with node strength. **(A)** Node strength for TDC vs. ADHD-C shows strong differentiation in regions of the medial prefrontal cortex among other distributed systems. **(B)** Node strength for TDC vs. ADHD-I shows differentiation distributed throughout the cortex as well, with prominent nodes including bilateral dorsolateral prefrontal, and cerebellar regions among others. **(C)** Comparisons between the subtypes show similar trends. [Node colors represent network categorization from a community detection procedure performed for a previous report (Dosenbach et al., 2010). Red—default; blue—cerebellum; yellow—fronto-parietal; black—cingulo-opercular; green—occipital; cyan—sensorimotor].

**Figure A4:**
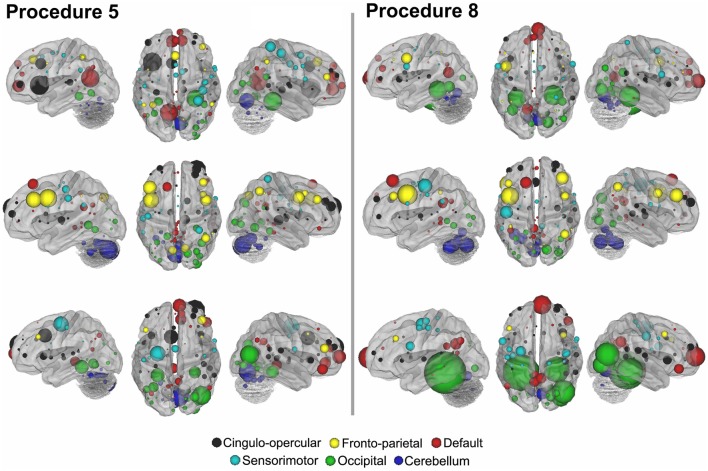
***Post-hoc* comparisons on the consensus features from the 2-group classification for Procedures 5 and 8.** While not identical, patterns across all of these movement correction procedures is largely consistent. **Top:** TDC vs. ADHD-C. **Middle:** TDC vs. ADHD-I. **Bottom:** ADHD-C vs. ADHD-I. **Left:** Procedure 5. **Right:** Procedure 8 [Node colors represent network categorization stemming from a community detection procedure performed for a previous report (Dosenbach et al., 2010). Red—default; blue—cerebellum; yellow—fronto-parietal; black—cingulo-opercular; green—occipital; cyan—sensorimotor].

**Figure A5:**
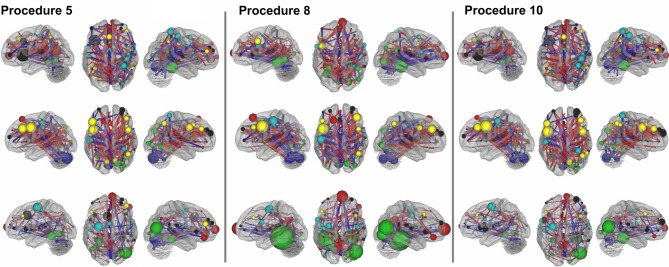
**As in Figures 8 and A4, *Post-hoc* comparisons on the consensus features from the 3-group classification.** Included are the vectors, which are used to calculate node strength. **Top:** TDC vs. ADHD-C. **Middle:** TDC vs. ADHD-I. **Bottom:** ADHD-C vs. ADHD-I. **Left:** Procedure 5. **Middle:** Procedure 8. **Right:** Procedure 10.

**Figure A6:**
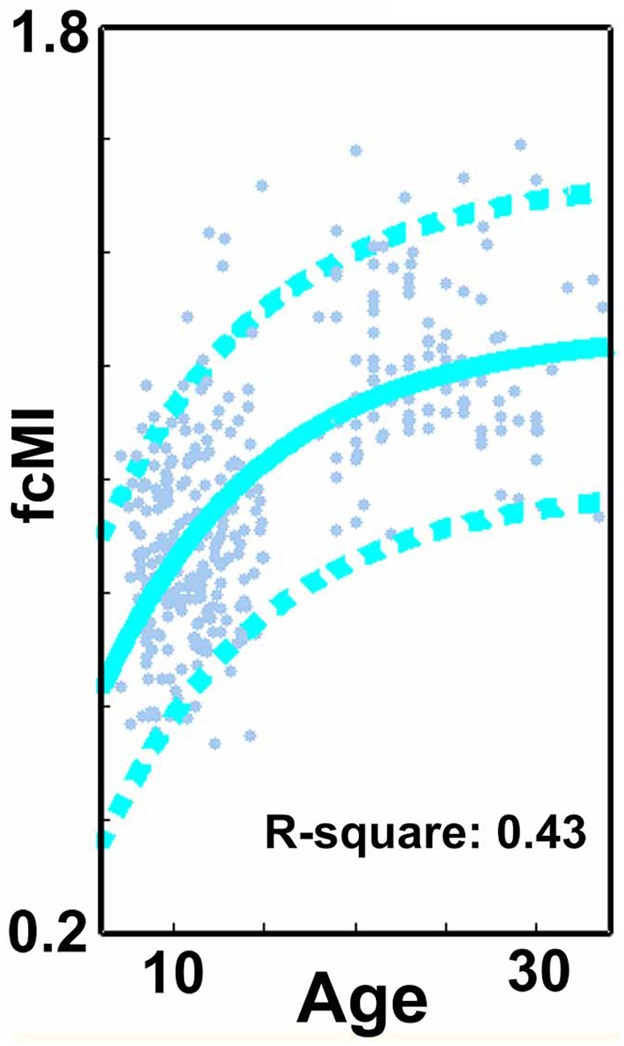
**A re-examination of SVR-based MVPA brain maturation curve for Procedure 7 (frame removal) while including only those subjects with <60% of their frames removed**.

For procedure 10, visualization of those nodes whose strength most strongly separated each of the two ADHD subtypes considered from TDC (i.e., Node Strength), revealed stark differences between the two (Figure 8; Figures A4, A5; Table A1). Nodes most strongly differentiating ADHD-C and TDC were distributed across the cortex (Figure 8), but were most prominent in the medial prefrontal and posterior parietal nodes of the default network. Other atypical regions included nodes of the sensorimotor, visual, and cingulo-opercular systems. Findings with regard to consensus features and ADHD-I were similar to those of ADHD-C in that they were largely distributed across systems including prominent features in the sensorimotor systems; however, the patterns between the subtypes were also quite distinct. In the case of ADHD-I, atypical nodes in left and right dorsolateral prefrontal regions along with the cerebellum were more strongly predictive. These particular nodes (see Figure A6) have been empirically linked with community detection methods to fronto-parietal systems (black/yellow) (Dosenbach et al., 2010) In addition, similar to ADHD-C, sensorimotor regions (light blue) (Figure 8) were also atypical in ADHD-I. The direct comparison between ADHD-I and ADHD-C were largely consistent with the distinctions noted above (Figure 8). As can be seen in Figure A4, procedures 8 and 10 revealed largely consistent findings with regard to overall patterns.

## Discussion

Consistent with recent work demonstrating the utility of data aggregation (Biswal et al., 2010), and in the spirit of the ADHD-200 Global Competition (ADHD-200-Consortium, 2012), we used the large-scale aggregate ADHD-200 dataset to highlight the need for stringent micro-movement correction. The work also provides the first insights into common and distinct neural substrates of the two most common ADHD subtypes. Application of graph-theoretic metrics implicated atypical connectivity across multiple systems in the combined subtype (ADHD-C), particularly in the default network. Although similar observations were present in the predominantly inattentive subtype (ADHD-I) functional connectivity differences were more evident in bilateral dorsolateral prefrontal regions, and in the cerebellum, rather than in the default mode network. Classification analyses aimed at predicting individual diagnostic status based on patterns of functional connectivity further highlight differential neural substrates for the two subtypes, and serve to provide a vision for future clinical applications of neuroimaging.

### Motion correction improves ADHD characterization

There were several notable findings regarding motion correction. For example, we showed that the relationship between movement measurements based on traditional reference frame calculations can, in some instances, be misleading. In particular, the relationship between traditional movement measurements and the BOLD signal is markedly non-linear. As such, traditional functional connectivity and fMRI movement correction that utilize linear regression are likely to be limited with regard to micro-movement correction. Importantly, however, while frame-to-frame measurements (i.e., FD), appear to be superior with relation to the assumption of linearity, they too share a non-linear relationship with the BOLD signal. Thus, simple linear regression of these measurements from the BOLD signal also provides only limited improvement with regard to motion artifacts (Figure 4). With that said, we found several methods that appeared relatively robust at controlling for motion artifact in the BOLD signal. All of these additional approaches reduced the distinction between short- and long-range connections in terms of their relationships with age.

Age-related growth curves were improved with several of the motion correction procedures, with the strongest age-related effects occurring when using DVARS and matching as the surrogate measure for frame removal (Figure 3). Nevertheless, there are some concerns with approaches that require frame removal. In particular, the approaches reduce sample size due to “matching” (i.e., subject exclusion) or, effectively removing subjects due to the high percentage of frames removed. Thus, statistical power is reduced. The large sample size here afforded us the ability to examine these conservative approaches, but other samples may not support the same opportunity.

Another concern relates to the potentially undesirable effects of frame removal on time-series analyses. Our simulations suggest that the correlation structure can be altered with frame removal; however, the concern appears to be largest only after significant portions of the frames are removed. One potential way to overcome this limitation for the frame removal procedures would be to interpolate missing values. While in theory we agree with this suggestion, our simulations suggest that the introduction of interpolation as a means of minimizing data-loss can be counter-productive, unless more elaborate approaches are developed and validated beyond the common methods attempted here [e.g., Carp (2011)]. Another alternative to avoid frame removal is provided with Procedure 10. This procedure utilized censoring techniques to generate a polynomial capable of correcting the original *r*-values, and was quite productive at motion correction. The results were promising, but the method is not able to completely resolve the issues noted by frame removal. The method still relies on a valid estimate of the correlation structure after removing frames, and thus still requires participant removal when a high degree of motion is present.

The use of mean movement parameter (i.e., mean FD) as a covariate in the group analyses also appeared to be a productive method to reduce motion-related artifact in the BOLD signal. This procedure was similar to that originally proposed by Van Dijk et al. (2012). The results using this procedure also show a significant reduction in short- and long-range indices, albeit less than frame censoring methods (see Figures 2 and 4). Importantly, the method does not suffer from the inherent reduction of power or generalizability from decreasing sample size.

Nonetheless, the three techniques reveal similar, albeit not identical findings. It should be emphasized, however, that while these approaches appear to perform respectably for reducing artifact induced by changes in head position, work in this area is currently ongoing and even more robust procedures are likely to emerge.

**Figure A7:**
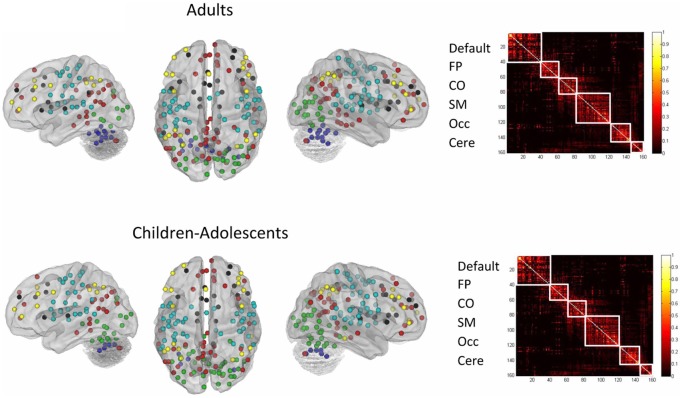
**Network categorization of each ROI was based on labels designated in a previous report (Dosenbach et al., 2010).** This categorization stemmed from a community detection procedure conducted on combined correlation matrices across adult subjects [e.g., see Fair et al. (2009)]. The modularity optimization algorithm of Newman was used (Newman, 2006). The modules (i.e., communities) used to categorize regions had a high quality index (Q) and were the most resistant to perturbation by randomization, measured by variation of information (VOI) (Karrer et al., 2008). These community assignments were re-examined here for both children and adults after applying procedure 8. In this instance, the weight conserving community detection algorithm used was based on the work by Rubinov and Sporns (2011). Network assignments largely agreed with the previous report and were similar in children and adults. On the left network assignments are visualized on the brain and on the right in matrix form.

**Figure A8:**
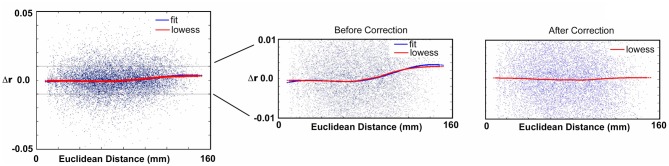
**Sample corrected subject data for Procedure 10.** For a given subject, correlation matrices are re-created after censoring (i.e., scrubbing). Differences in the correlation values from the original matrix and the censored matrix are then plotted as a function of distance. The red curve shows the trend of delta *r* based on LOWESS smoothing. The blue curve shows the polynomial (see Methods) fit. The method is applied on each subject individually such that every subject will have a fit for their respective curve. The fit is then used to regress the effect of movement on the original *r*-values. Censoring the frames as in Procedure 7 after applying this procedure reveals no effect of movement (Right panel).

Similar to a recent report (Satterthwaite et al., 2012), our findings show that many of the developmental principles identified previously are still present even after stringent motion correction, while others are not. For example, age-related predictions are still possible and were even improved with motion correction procedures (Dosenbach et al., 2010). However, while the changes in short- and long-range connectivity appear to remain, this phenomena is significantly reduced after motion correction procedures (and becomes only weakly significant for some procedures)—highlighting that the distance between those connections that get weaker with age and those that get stronger overlap more than was previously appreciated. Network configuration, while not identical, appears to be similar to that of adults, even in our younger ages, as shown in Figure A7 and in Power et al. (2012a). Thus, while significant changes do occur throughout development, the general network structure appears to be intact at early ages. It is possible that similar findings will unfold for the aging literature as well (Meunier et al., 2009; Van Dijk et al., 2012). Most importantly, however, these data in aggregate highlight the need to take careful consideration of movement related artifact in all connectivity studies, particularly during classification of clinical samples as noted below.

### The functional neurobiology of ADHD-C and ADHD-I is distinct

Over the course of the last decade, the validity of DSM-IV subtype classifications has been repeatedly challenged. Combining statistical analysis with a variety of neuropsychological, behavioral and clinical observation approaches, some have questioned the validity of distinguishing between combined and inattentive subtypes, while others have called for considerations of further subgroupings [for a review see Willcutt et al. (2012)]. Until now, neuroimaging studies have contributed little to this debate—largely for lack of sufficient data. Using rs-fcMRI, we found that while commonalities exist in the neural substrates of the two subtypes, distinctions are also present—suggesting that ADHD-I may capture not simply a “less severe” form of ADHD-C, but a neurobiologically distinct syndrome. This reinforces the conclusion from Willcutt et al. (2012) that additional neuroimaging data on the question of ADHD subtypes is sorely needed. It will remain to be seen whether longitudinal brain imaging data can help resolve the problem of subtype instability (Lahey and Willcutt, 2010) in the disorder.

Consistent with recent work emphasizing the value of network-based approaches for understanding psychiatric illnesses such as ADHD (Castellanos et al., 2008; Fair et al., 2010; Costa Dias et al., 2012; Mills et al., 2012), observed differences among the subtypes can be understood in terms of their network distributions. Findings for both subtypes were relatively distributed, with each affecting only a subset of regions in any one network. However, most notable in the ADHD-I group was the prominence of differences in specific regions of the fronto-parietal and cerebellar systems—specifically dorsolateral prefrontal regions. The fronto-parietal network is believed to be an important system for task level control (Dosenbach et al., 2008). Given that ADHD-I subtype symptoms are predominantly those of inattention, this finding is largely consistent with the idea that this subtype is characterized mainly by problems in task level control systems. While both subtypes revealed largely distributed atypical connectivity, findings for the ADHD-C were more prominent in the default network. These findings for ADHD-C are consistent with a growing body of studies implicating the default network in ADHD (Castellanos et al., 2008; Fassbender et al., 2009; Fair et al., 2010), and is intriguing given a growing number of studies highlighting the motivational as well as affective aspects of the disorder (Sagvolden et al., 2005; Musser et al., 2011) [for a reviews see Nigg and Casey (2005); Castellanos et al. (2006)]. In particular, impairments in incentive salience, motivation, and reward processing are increasingly appreciated in ADHD. While speculative, it should be noted that key components of the functional neuroanatomy of these processes are based within the default network (e.g., ventral striatum, ventromedial cortex). Recent theories suggest the default network is associated with remembering the past, as well as planning for, and anticipating future events (Buckner and Carroll, 2007; Buckner et al., 2008). As stated by Buckner and colleagues, the default network may support a “set of processes by which mental simulations are used adaptively to imagine events beyond those that emerge from the immediate environment.” Analogous to this notion, the nature of “motivational”-based theories is that children with ADHD are unable to correctly explore, anticipate, and “value” outcomes between present action and future rewards. If the default network is indeed important for using past experiences to explore and anticipate future events, one might anticipate that aberrations in this network and its links to other systems would map onto the ADHD phenotype and, in particular, the impulsive and hyperactive behavior of the ADHD-C subtype, as found here. Studies of gray matter thickness (Shaw et al., 2007) and connectivity measurements (Shannon et al., 2011) have shown that many of these same regions correspond to various forms of impulsivity measures, supporting this claim.

Along with the other systems noted above, ADHD-associated differences in connectivity of primary sensorimotor systems was observed across both subtypes. Children with ADHD often demonstrate difficulties with motor control, paralleling difficulties with higher-order executive control (Pennington and Ozonoff, 1996; Mostofsky et al., 2003). A consistent characteristic of children with ADHD is that they fail to meet age-norms on timed repetitive and sequential movements and manifest a greater amount of motor overflow than age-matched controls (Denckla and Rudel, 1978; Mostofsky et al., 2003). Furthermore, in parallel with numerous recent studies showing ADHD-associated increases in intrasubject response time variability (Castellanos et al., 2005; Vaurio et al., 2009; Epstein et al., 2011), children with ADHD show increased variability during performance of simple motor skills and during motor adaptation (Izawa et al., 2012). Both overflow and variability in motor execution have been found to be related to ADHD-associated impairments at the behavioral level (Mostofsky et al., 2003). Converging evidence from behavioral, imaging, and electrophysiologic studies suggest that ADHD is associated with dysfunction across multiple parallel frontal-subcortical circuits and while it may not be the case that ADHD is a primary motor disorder, it does seems likely that core ADHD impairments are reflected in motor function in practically measurable and biologically meaningful ways (Mostofsky and Simmonds, 2008). To that end, recent studies of motor cortex physiology using Transcranial Magnetic Stimulation (TMS) reveal that subjects with ADHD show reduced Short Interval Cortical Inhibition (SICI) and that lower SICI is robustly correlated with parent ratings of more severe hyperactive/impulsive and inattentive behavior (Gilbert et al., 2006). The findings from the current study further suggest that patterns of sensorimotor connectivity may prove effective in identifying ADHD, particularly those with the combined subtype.

In summary, our findings demonstrate the potential utility of functional MRI approaches in characterizing clinical heterogeneity in ADHD, which should be similarly useful with other psychiatric disorders. While the present work focused on testing the existing categorization among individuals with ADHD, the previous work highlighted above and the distributed nature of our current findings is likely suggestive of significantly more heterogeneity in this population. Future work is likely to benefit from stronger, perhaps more homogenous subgroupings via the application of data-driven methods that identify clinical subtypes based upon variation in the patterns of neuropsychological measures (Fair et al., 2012) or brain connectivity itself. Additionally, given clinical observations that children with ADHD-C over the course of development later present as ADHD-I based on currend diagnostic measures, future work may benefit from comparison of “converters” with those who were diagnosed with ADHD-I throughout their childhood. Such endeavors will undoubtedly require the acquisition of large and tightly coordinated data acquisition across centers.

### Toward imaging-based classification of diagnostic status

Beyond a simple characterization of the neural signatures of ADHD subtypes, the findings presented here highlight the translational potential of rs-fcMRI for developmental neuropsychiatric disorders in general by demonstrating the ability of SVM-based MVPA to classify individuals with the disorder using the rs-fcMRI. This demonstration was accomplished even after our most conservative motion correction procedures. The work provides several important outcomes, and suggests that there is an ability to classify individuals based on disease status using information available from a brief MRI brain scan. While this particular effort did not focus on maximizing our supervised learning algorithm and feature selection, we were able to make significant disease status predictions well above chance.

We anticipate classification rates with the current ADHD-200 sample will continue to improve with optimization of supervised and unsupervised learning approaches. Optimization and extension of feature sets to include other potential markers is also likely to enhance classification. However, it is only through the future creation of a large-scale datasets, with coordinated recruitment, deep phenotyping, multimodal data acquisition (e.g., rs-fcMRI, diffusion imaging, ASL), and likely improved homogeneity in our subgrouping (Fair et al., 2012) that a fair assessment of the predictive potential of MR-based approaches will be realized. Inclusion of complementary measures from non-MR modalities such as EEG and genetics may further enhance the completeness and accuracy of predictive models.

## Limitations

The findings of the present work should be considered in light of several cautions. Notably, while our movement correction procedures were carefully considered, there is still room for improvement in controlling for this confound. In addition, the present work made use of data aggregated across imaging sites that were not coordinated with respect to their recruitment, diagnostic, or imaging protocols—as such, marked site-related variation exists. Our findings suggest that despite potential differences across centers, there are brain features related to the presence of ADHD and ADHD subtype that are robust to this variation. However, future studies with tightly coordinated imaging and phenotypic data acquisition will be required to replicate the findings of present work and identify additional ADHD-related features that may have been overlooked in the present work due to site-related variation. We note that ADHD relies on strict symptom counts and the DSM-IV system for identifying ADHD-I includes participants who are subthreshold for hyperactivity. Thus, future work may consider examining the extremes (e.g., <3 hyperactive symptoms for ADHD-I) in order to avoid confounds with regard to the imprecise nature of the diagnostic system. Last, we mention that this work does not consider the possibility that the differences noted here could be unique to the effort required to remain still in the ADHD population. Nonetheless, the ability to make valid predictions in individuals with atypical functional neuroanatomy using rs-fcMRI data acquired across multiple institutions provides evidence that this approach can be fruitfully applied in translational studies of disorders with developmental origins.

## Conclusions

Taken together, these results suggest that ADHD-I has distributed atypical connectivity, with prominent findings in control systems, which may underlie the prominent inattentive symptoms in this population. ADHD-C also shows distributed atypical connectivity, with prominent findings in systems such as the default network, which may mark for an inability to exert top-down attentional control—perhaps in the context of an inability to integrate contextual information supported by default mode processing. Both populations showed atypical nodes in primary sensorimotor systems, supporting previous work implicating this system in ADHD (Mostofsky et al., 2006). These findings point to potential distinct connectivity patterns underlying ADHD subtypes, but also emphasizes the vast heterogeneity in these populations, which will need to be considered in future clinical investigations of ADHD.

### Conflict of interest statement

The authors declare that the research was conducted in the absence of any commercial or financial relationships that could be construed as a potential conflict of interest.

## Appendix

### Participants and measures

Data from Brown University, Beijing Normal University, Kennedy Krieger Institute, NYU Child Study Center, and Oregon Health and Science University were aggregated for youth aged 7–14 years. The resulting dataset comprised of 455 control subjects and 193 subjects with a DSM-IV-TR diagnosis of ADHD. A summary of the primary demographics for each sample is provided in Table 1. Informed written consent or assent was obtained for all participants, and procedures complied with the Human Investigation Review Board at respective universities. As data for this investigation were aggregated retrospectively (as a large collaborative effort), slightly different ADHD assessment protocols were used across institutions. These procedures are detailed below.

#### Oregon Health and Science University

Psychiatric diagnoses were based on evaluations with the *Kiddie Schedule for Affective Disorders and Schizophrenia*—KSADS-I (Puig-Antich and Ryan, 1986; Kaufman et al., 1997) administered to a parent; parent and teacher (short form) *Conners' Rating Scale-3rd Edition* (Conners, 2008); and a clinical review by a child psychiatrist and neuropsychologist who had to agree on the diagnosis. Estimates of intelligence were evaluated with a three-subtest short form (Block Design, Vocabulary, and Information) of the *Wechsler Intelligence Scale for Children, Fourth Edition* (WISC-IV) (Wechsler, 1999, 2003).

Children (7–11 years) were excluded if they did not meet criteria for ADHD or non-ADHD groups (i.e., children deemed sub-threshold by the clinicians were excluded). Children were also excluded for an IQ < 80, if a history of neurological illness, chronic medical problems, sensorimotor handicap, autistic disorder, mental retardation, or significant head trauma (with loss of consciousness) was identified by parent report, or if they had evidence of psychotic disorder or bipolar disorder on the structured parent psychiatric interview. Children prescribed short-acting stimulant medications were scanned after a minimum washout of five half-lives (i.e., 24–48 h depending on the preparation). Typically developing control children (TDC) were excluded for presence of conduct disorder, major depressive disorder, or history of psychotic disorder, as well as for presence of ADHD. All children were right handed.

#### Kennedy Krieger Institute

Psychiatric diagnoses were based on evaluations with the Diagnostic Interview for Children and Adolescents, Fourth Edition [DICA-IV(Reich et al., 1997)], a structured parent interview based on DSM-IV criteria. Estimates of intelligence were evaluated with the WISC-IV (Wechsler, 1999, 2003) and academic achievement was assessed with the Wechsler Individual Achievement Test-II.

All study participants were between 8.0 and 13.0 years, and had a Full Scale IQ of 80 or higher. Children with a specific language disorder or a Reading Disability (RD) were either screened out before a visit or based on school assessment completed within 1 year of participation. RD was based on a statistically significant discrepancy between a child's FSIQ score and his/her Word Reading subtest score from the Wechsler Individual Achievement Test-IIa, or a standard score below 85 on the Word Reading subtest, regardless of IQ score. Participants with visual or hearing impairment, or history of other neurological or psychiatric disorder were excluded.

Children assigned to the ADHD group met criteria for ADHD on the DICA-IV and either had: (1) a T-score of 65 or greater on Conners' Parent Rating Scale-Revised, Long Form [CPRS-R; (Epstein et al., 1997)] subscales “L” (DSM-IV Inattentive) and/or “M” (DSM-IV Hyperactive/Impulsive), or (2) met criteria on the DuPaul ADHD Rating Scale IV (DuPaul et al., 1998) with six out of nine items scored 2 or higher from Inattention items and/or six out of nine scored 2 or higher from the Hyperactivity/Impulsivity items. Children with comorbid DSM-IV diagnoses other than Oppositional Defiant Disorder or Specific Phobias were excluded. DSM-IV criteria and the aforementioned rating scales were also used to evaluate the three ADHD subtypes (Inattentive: ADHD-I; Hyperactive/Impulsive: ADHD-HI; Combined: ADHD-C). Children with ADHD were assigned to the ADHD-I group if they met criteria for inattentiveness but not hyperactivity/impulsivity on the DICA-IV, and had a T-score of 65 or greater on the CPRS Scale L, and a T-score of 60 or less on the CPRS Scale, or had a rating of 2 or 3 on six out of nine Inattention items on the ADHD Rating Scale IV and a rating of 2 or 3 on four or fewer items on the Hyperactivity/Impulsivity scale. Children were assigned to the ADHD-HI if they met criteria for hyperactivity/impulsivity but not inattention on the DICA-IV, and a T-score of 65 or greater on the CPRS Scale M and a T-score of 60 or less on the CPRS Scale L, or had a rating of 2 or 3 on six out of nine Hyperactivity/Impulsivity items on the ADHD Rating Scale IV and a rating of 2 or 3 on four or fewer items on the Inattention scale. All other children who met criteria for ADHD were assigned to the ADHD-C (Combined subtype) group. Children with ADHD taking psychoactive medications other than stimulants were excluded. Children who were taking stimulant medication were asked to withdraw from these medications the day before and the day of testing/scanning.

TDC participants were required to have T-scores of 60 or below on the DSM-IV Inattention (L) and DSM-IV Hyperactivity (M) subscales of CPRS-R and no history of behavioral, emotional, or serious medical problems. Additionally, TDC individuals were not included if there was a history of school-based intervention services as established by parent interview, or if they met DSM-IV psychiatric disorder except specific phobia as reported on the DICA-IV.

#### New York University

Psychiatric diagnoses were based on evaluations with the Schedule of Affective Disorders and Schizophrenia for Children—Present and Lifetime Version (KSADS-PL) administered to parents and children and the CPRS-LV (Epstein et al., 1997). Intelligence was evaluated with the Wechsler Abbreviated Scale of Intelligence (WASI) (Wechsler, 1999, 2003). Inclusion in the ADHD group required a diagnosis of ADHD based on parent and child responses to the KSADS-PL as well as on a T-score greater than or equal to 60 on at least one ADHD related index of the CPRS-R: LV. ADHD subtype identification was based on interview and review of available records. Psychiatric comorbidity was not exclusionary except for Conduct Disorder, Bipolar and Major Depressive Disorders, as well as any psychotic disorders. Psychostimulant drugs were withheld at least 24 h before scanning. Inclusion criteria for TDC required absence of any Axis-I psychiatric diagnoses per parent and child KSADS-PL interview, as well as T-scores below 60 for all the CPRS-R: LV ADHD summary scales. Estimates of FSIQ above 80, right-handedness and absence of other chronic medical conditions were required for all children.

Table A1 **Node strength values represented in Figures 7 and 8**.

#### Peking University/Beijing Normal University

Study participants with the diagnosis of ADHD were initially identified using the Computerized Diagnostic Interview Schedule IV (C-DIS-IV) (Shaffer et al., 2000). Upon referral for participation to the study participation, all participants (ADHD and TDC) were evaluated with the Schedule of Affective Disorders and Schizophrenia for Children—Present and Lifetime Version (KSADS-PL) with one parent for the establishment of the diagnosis for study inclusion. Thus, identification of the ADHD subtype was based on this psychiatric interview. Additional inclusion included: (1) right-handedness, (2) no history of neurological disease and no diagnosis of either schizophrenia, affective disorder, or pervasive development disorder, and (3) full scale Wechsler Intelligence Scale for Chinese Children-Revised (WISCC-R) score of greater than 80. Psychostimulant medications were withheld at least 48 h prior to scanning. Informed consent was also obtained from the parent of each subject and all of the children agreed to participate in the study.

#### Brown University and Bradley Hospital

Psychiatric diagnoses were based on evaluation by the same board-certified child/adolescent psychiatrist (DPD) for all participants, using the Child Schedule for Affective Disorders Present and Lifetime version (KSADS-PL) administered to parents and children separately (Puig-Antich and Ryan, 1986). All participants completed the WASI as an overall measure of cognitive ability (Wechsler, 1999, 2003). Children in the ADHD group had to meet Diagnostic and Statistical Manual 4th Edition Text Revision (DSM-IV-TR) criteria for ADHD, as determined by parent and child answers to the KSADS-PL and were required to have ongoing psychiatric treatment. Exclusion criteria were comorbid mood or anxiety disorders, any autism spectrum disorder, medical illness that was unstable, or could cause psychiatric symptoms, or substance abuse within ≤2 months of participation. All ADHD participants taking psychostimulant medications (i.e., derivatives of methylphenidate or dextroamphetamine) were scanned when medication-free for five drug half-lives. TDC participant inclusion criteria were a negative history of psychiatric illness in the participant and their first-degree relatives. Exclusion criteria were pregnancy, ongoing medical or neurological illness or past/present psychiatric, or substance disorder. All participants had an IQ greater than 70.

### Data acquisition and processing

#### Oregon Health and Science University

Participants were scanned using a 3.0 Tesla Siemens Magnetom Tim Trio scanner (Siemens, Erlangen, Germany) with a 12-channel head-coil at the OHSU Advanced Imaging Research Center. One high-resolution T1-weighted MPRAGE sequence lasting 9 min and 14 s (TR = 2300 ms, TE = 3.58 ms, orientation = sagittal, 256 × 256 matrix, resolution = 1^3^ mm) was collected. Blood-oxygen level dependent (BOLD)-weighted functional imaging data were collected in an oblique plane (parallel to the ACPC) using T2^*^-weighted echo-planar imaging (EPI) (TR = 2500 ms, TE = 30 ms, flip angle = 90°, FOV = 240 mm, 36 slices covering the whole brain, slice thickness = 3.8 mm, in-plane resolution = 3.8 × 3.8 mm). Steady state magnetization was assumed after five frames (~10 s). Three runs of 3.5 min each were obtained. During rest periods subjects were instructed to stay still, and fixate on a standard fixation-cross in the center of the display.

#### Kennedy Krieger Institute

Participants were scanned using a 3.0 Tesla Philips scanner with an eight-channel head-coil. One high-resolution T1-weighted MPRAGE sequence (TR = 7.99 ms, TE = 3.76 ms, flip angle = 8°) was collected. BOLD-weighted functional imaging data were collected using T2^*^-weighted EPI (TR = 2500 ms, TE = 30 ms, flip angle = 75°, 2D-SENSE EPI). The run lasted either 5 min 20 s or 6 min 30 s. During rest participants were instructed to relax, stay as still as possible, keep eyes open, and fixate on a center cross.

#### New York University

Participants were scanned using a Siemens Allegra 3.0 Tesla scanner at the NYU Center for Brain Imaging. For each participant a T1-weighted MPRAGE sequence was acquired using a magnetization prepared gradient echo sequence (TR = 2530 ms; TE = 3.25 ms; TI = 1100 ms; flip angle = 7°; 128 slices; FOV = 256 mm; acquisition voxel size = 1.31 × 1.3 mm). A 6-minute resting scan comprising 180 contiguous whole-brain functional volumes was also acquired for each participant using a multi-echo EPI sequence (TR = 2000 ms; flip angle = 90°; 33 slices; voxel size = 3 × 3 × 4 mm; effective TE = 30 ms, FOV = 240 × 192 mm). During rest periods participants were instructed to lie still and relax with their eyes open, while a standard fixation-cross was presented in the center of the display.

#### Peking University Samples

***Dataset #1***. Images were acquired using a Siemens Trio 3.0 Tesla scanner in National Key Laboratory of Cognitive Neuroscience and Learning, Beijing Normal University. For each participant, a high-resolution T1-weighted anatomical image was acquired [128 sagittal slices, slice thickness/gap = 1.33/0 mm, in-plane resolution = 256 × 192, TR = 2530 ms, TE = 3.39 ms, inversion time (TI) = 1100 ms, flip angle = 7°, FOV = 256 × 256 mm^2^]. A resting-state scan was obtained for each participant (33 axial slices, TR = 2000 ms, TE = 30 ms, flip angle = 90°, thickness/gap = 3.5/0.7 mm, FOV = 200 × 200 mm^2^, matrix = 64 × 64, 240 volumes), as well as diffusion tensor imaging (not reported here).

***Dataset #2***. Images were acquired using a Siemens Trio 3.0 Tesla scanner in National Key Laboratory of Cognitive Neuroscience and Learning, Beijing Normal University. All of the resting-state functional data were acquired using an EPI sequence with the following parameters: 33 axial slices, TR = 2000 ms, TE = 30 ms, flip angle = 90°, slice thickness/skip = 3.0/0.6 mm, FOV = 200 × 20 mm, in-plane resolution = 64 × 64, 240 volumes. For each patient, T1-weighted structural images were acquired using a spoiled gradient-recalled sequence covering the whole brain and used for the purpose of image registration (see Data preprocessing). The T1-weighted structural images were acquired with the following parameters: 176 sagittal slices, TR = 2530 ms, TE = 3.45 ms, flip angle = 7°, slice thickness/skip = 1.0/0 mm, FOV = 256 × 256 mm, in-plane resolution = 256 × 256.

Children with ADHD were scanned twice, in a double-blinded, randomized, counterbalanced way. The two scans were at least 2 days apart, and each scan was taken 1 h after either 10 mg MPH administration or placebo (Vitamin B6, 10 mg). All the patients had not received stimulant treatment for at least 2 days before the first scan, and were asked not to take any stimulant between two scans. The control boys were scanned once without MPH or placebo taken for ethical reasons. Only placebo scans were used for the present study.

***Dataset #3***. This dataset was previously employed in a prior study (Cao et al., 2009).

Images were acquired using a Siemens Trio 3.0 Tesla scanner in the Institute of Biophysics, Chinese Academy of Sciences. Rest scans were acquired using an EPI sequence with the following parameters: 30 axial slices, TR = 2000 ms, TE = 30 ms, flip angle = 90°, thickness/skip = 4.5/0 mm, FOV = 220 × 220 mm, matrix = 64 × 64, 240 volumes. Participants were asked simply to remain still, close their eyes, think of nothing systematically, and not fall asleep. Additionally, for each participant, a high-resolution T1-weighted anatomical image using a spoiled gradient-recalled sequence covering the whole brain was acquired. The data were collected in a period of about 2 years and some modifications were made in the sequence of the structural images. Most of the subjects (see details below) were scanned with one of the following two kinds of parameters: (1) 192 slices, TR = 2000 ms, TE = 3.67 ms, inversion time = 1100 ms, flip angle = 12°, FOV = 240 × 240 mm, matrix = 256 × 256, used in 8 patients and 12 controls; (2) 176 slices, TR = 1770 ms, TE = 3.92 ms, inversion time = 1100 ms, flip angle = 12°, FOV = 256 × 256 mm, matrix = 512 × 512, used in 9 patients and 11 controls. Other scanning sessions, which have no relation to the present study, are not described here.

#### Brown University

Scans were acquired on a Siemens Tim Trio 3.0 Tesla scanner with a 12-channel head-coil. A high-resolution T1-weighted MPRAGE anatomical image was acquired for normalization and localization (TR = 2250 ms, TE = 2.98 ms, T1 = 900 ms, flip angle = 9°, slices = 160, FOV = 256 mm, voxels = 1 × 1 × 1 mm). The resting-state functional connectivity scan contained 256 continuous BOLD volumes (TR = 2000 ms, TE = 25 ms, flip angle = 90°, slices = 35, FOV = 192 mm, voxels = 3 × 3 × 3 mm). The scan lasted for 8 min and 36 s. During the scan, participants were instructed to rest with their eyes open while the word “relax” was back-projected via LCD projector.

#### Washington University

Scans were acquired on a Siemens Tim Trio 3.0 T Scanner with a Siemens 12-channel Head Matrix Coil. A T1-weighted sagittal MP-RAGE anatomical image was acquired (TE = 3.06 ms, TR-partition = 2.4 s, TI = 1000 ms, flip angle = 8°, 176 slices with 1 × 1 × 1 mm voxels). The resting-state functional connectivity scan were obtained using a BOLD contrast sensitive gradient echo echo-planar sequence (TE = 27 ms, flip angle = 90°, in-plane resolution = 4 × 4 mm; volume TR = 2.5 s). Whole-brain coverage for the functional data was obtained using 32 contiguous interleaved 4 mm axial slices.
